# A comprehensive toxicological analysis of *trans*-fatty acids (TFAs) reveals a pro-apoptotic action specific to industrial TFAs counteracted by polyunsaturated FAs

**DOI:** 10.1038/s41598-023-32083-9

**Published:** 2023-04-11

**Authors:** Yusuke Hirata, Naoki Kashiwabara, Yuki Nada, Aya Inoue, Emiko Sato, Takuya Noguchi, Atsushi Matsuzawa

**Affiliations:** 1 Laboratory of Health Chemistry, Graduate School of Pharmaceutical Sciences, Tohoku University, 6-3 Aoba, Aramaki, Aoba-Ku, Sendai, 980-8578 Japan; 2 Division of Clinical Pharmacology and Therapeutics, Graduate School of Pharmaceutical Sciences, Tohoku University, Sendai, Japan

**Keywords:** Biochemistry, Cell biology, Risk factors

## Abstract

*trans*-Fatty acids (TFAs) are unsaturated fatty acids containing at least one carbon–carbon double bond in *trans* configuration, which are classified into two groups according to their food source: industrial TFAs (iTFAs) and ruminant TFAs (rTFAs). Previous epidemiological evidence has demonstrated a preferential association of iTFAs, rather than rTFAs, with various diseases including cardiovascular diseases. However, it is still unknown how iTFAs exert their specific toxicity and what effective treatments are available to mitigate their toxicity. Here, we performed a comprehensive toxicological assessment of TFAs based on the toxicity mechanism that we established previously. We found that iTFAs including elaidic acid (EA), but not other types of fatty acids including rTFAs, had a strong pro-apoptotic effect upon treatment of extracellular ATP, a damage-associated molecular pattern that induces apoptosis through the apoptosis signal-regulating kinase 1 (ASK1)-p38 MAP kinase pathway. We also found that polyunsaturated fatty acids (PUFAs), such as docosahexaenoic acid (DHA), potently suppressed EA-dependent increase in ASK1 activation and apoptosis. These results demonstrate that iTFAs specifically exert toxicity by targeting ASK1, and that PUFAs serve as their effective suppressor. Our study provides a molecular basis for risk assessment of foods, and for new prevention and treatment strategies for TFA-related diseases.

## Introduction

**Figure 1:**
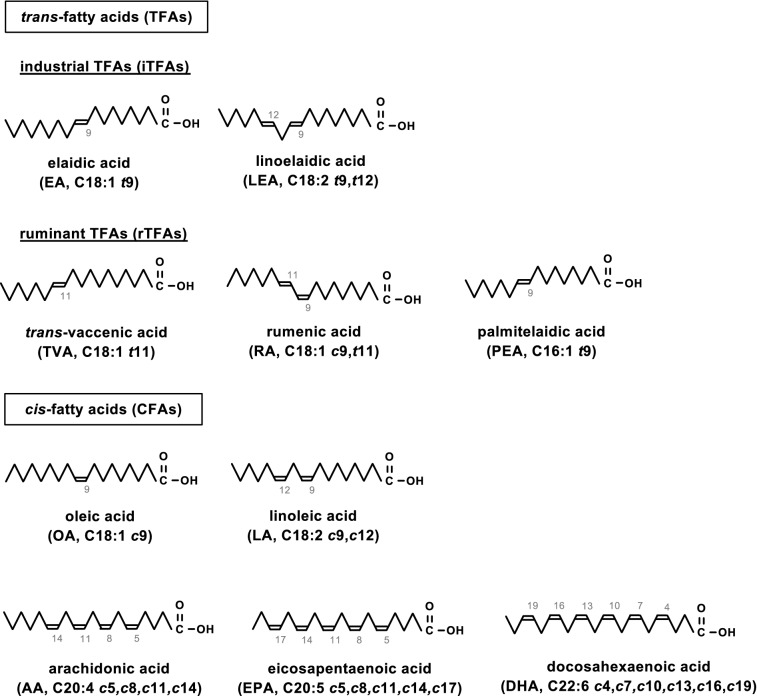
Structures of the fatty acids utilized in this study. The numbers in the figure represent the positions of carbon–carbon double bonds. In parentheses, the following information is shown: abbreviation, the number of carbon atoms, and the number, configuration (*t*, *trans*; *c*, *cis*) and positions of the carbon–carbon double bonds.

*trans*-Fatty acids (TFAs) are unsaturated fatty acids (UFAs) that possess one or more *trans* carbon–carbon double bonds. In the human body, fatty acid desaturating enzymes introduce only *cis* double bonds into fatty acids. Hence, whereas enzymatically synthesized UFAs contain all *cis* double bonds, hereafter referred to as *cis*-fatty acids (CFAs), TFAs are exclusively obtained from dietary sources^1^. According to the types of sources, TFAs are divided into two groups: industrial and ruminant. Industrial TFAs (iTFAs), such as elaidic acid (EA, C18:1 *t*9) and linoelaidic acid (LEA, C18:2 *t*9,*t*12), are generated as byproducts during food production processes, such as partial hydrogenation of edible oils containing CFAs, and are abundant in processed foods, including snacks and fast food products^2^. On the other hand, ruminant TFAs (rTFAs), such as *trans*-vaccenic acid (TVA, C18:1 *t*11), rumenic acid (RA, C18:2 *c*9*,t*11), and palmitelaidic acid (PEA, C16:1 *t*9), are mainly produced during bacterial biohydrogeneation of CFAs in the rumen of ruminants (e.g. cows and sheep), and are present in meat and dairy products (Fig. 1)^2^. Accumulating epidemiological evidence has demonstrated that the intake of TFAs, particularly iTFAs, raises the risk of various disorders, such as systemic inflammation, metabolic syndrome, neurodegenerative diseases (NDs), and cardiovascular diseases (CVDs)^3–7^, which has been supported by mouse model studies^8–10^. However, structure–toxicity relationships of TFAs have yet to be determined, since the molecular mechanism underlying TFA toxicity has remained elusive.

We have recently shown a novel toxicity mechanism of TFAs; TFAs promote inflammatory signaling and cell death in response to extracellular ATP (eATP), one of the damage-associated molecular patterns (DAMPs) that are known as potent pro-inflammatory factors, derived from injured cells^11,12^. eATP induces apoptosis through activation of the apoptosis signal-regulating kinase 1 (ASK1)-p38 mitogen activated protein (MAP) kinase pathway, initiated by reactive oxygen species (ROS) that are generated from NADPH oxidases downstream of the P2X purinoceptor 7 (P2X_7_)^13^. We showed that TFAs including EA, but not their corresponding *cis* isomers, potentiate eATP-induced activation of the ASK1-p38 pathway, and thereby promote apoptosis in macrophages and microglial cells^11,12^, which is closely associated with the pathogenesis of CVDs and ND, respectively. Besides these cell types, P2X_7_ receptor is expressed in diverse types of cells, such as epithelial cells and neuronal cells, and is involved in various diseases, including diabetes, obesity, NDs, and CVDs, through enhancing inflammatory responses and cell death^14,15^. Thus, our findings provided mechanistic insight into the etiology of TFA-related disorders.

In this study, based on our recently established toxicity mechanism of TFAs, we performed a comprehensive toxicity assessment of five major food-derived TFAs: EA and LEA as iTFAs, and TVA, RA, and PEA as rTFAs (Fig. 1)^16^. We showed that EA and LEA potently promote eATP-induced activation of ASK1 and p38, and ultimately cell death, whereas TVA, RA and PEA scarcely did, in consistent with the previous epidemiological studies showing a particular association of the iTFAs with various disorders including CVDs^6^. Moreover, by focusing on the ameliorating effect of all-*cis* poly-unsaturated fatty acids (PUFAs), such as eicosapentaenoic acid (EPA, C20:5 *c*5, *c*8, *c*11, *c*14, *c*17) and docosahexaenoic acid (DHA, C22:6 *c*4, *c*7, *c*10, *c*13, *c*16, *c*19), on various pathological conditions^17^, we found that co-treatment with PUFAs strongly blocked the pro-apoptotic action of iTFAs by suppressing ASK1 hyperactivation. These results demonstrate that iTFAs have a unique pro-apoptotic function during eATP stimulation, and that PUFAs serve as a potent suppressor for iTFA toxicity. Our study provides insight into developing new strategies for the prevention and treatment of TFA-related disorders.

## Results

### iTFAs specifically promote extracellular ATP-induced apoptosis signaling

**Figure 2:**
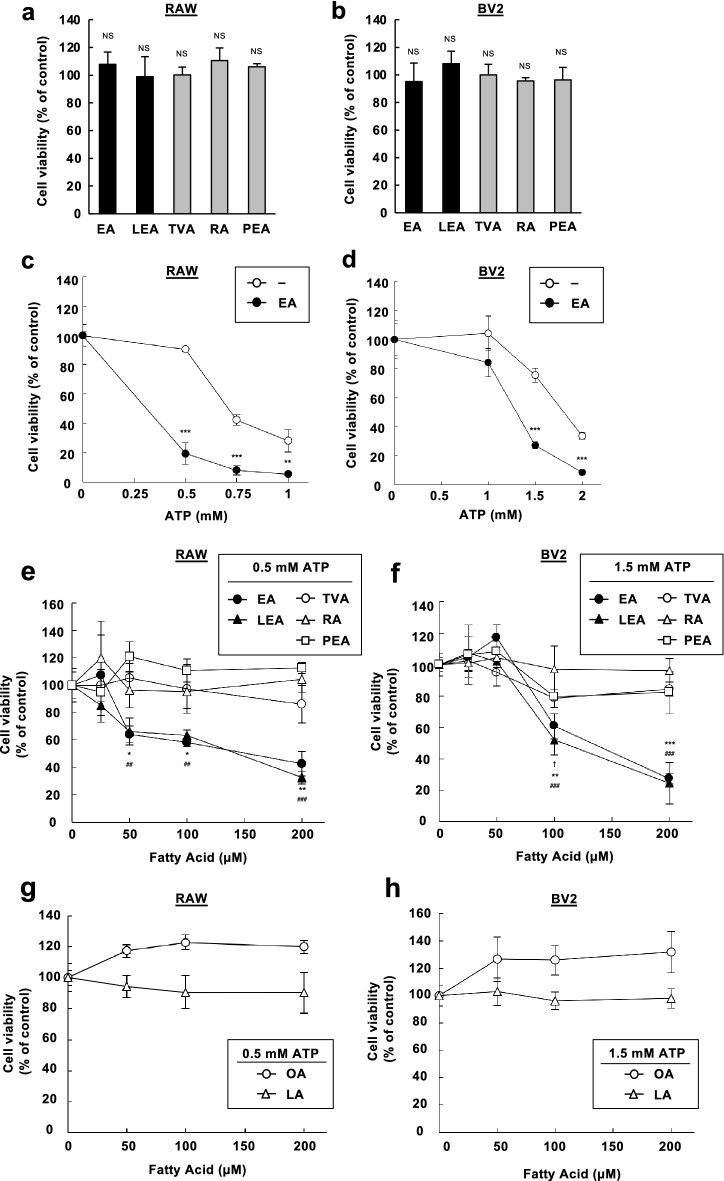
iTFAs specifically promote eATP-induced apoptosis. (**a**, **b**) RAW264.7 (**a**) and BV2 (**b**) cells were treated with the indicated TFAs for 20 h at 200 µM, and assayed for cell viability. Data are shown as mean ± SD (n = 3). NS: not significant (vs control without fatty acid). (**c**, **d**) RAW264.7 (**c**) and BV2 (**d**) cells were pretreated with 200 µM EA for 12 h, stimulated with various concentrations of ATP for 6 h, and assayed for cell viability. Data are shown as relative cell viability (mean ± SD, n = 3), normalized with the viability of cells stimulated with ATP without fatty acid. **p < 0.01; ***p < 0.001 (vs ATP-stimulated cells without EA). (**e**–**h**) RAW264.7 (**e**, **g**) and BV2 (**f**, **h**) cells were pretreated with the indicated concentrations of TFAs (**e**, **f**) or CFAs (**g**, **h**) for 12 h, treated with ATP at 0.5 mM (e, g) and 1.5 mM (**f**, **h**) for 6 h, and then assayed for cell viability. Data are shown as relative cell viability (mean ± SD, n = 3), normalized with the viability of cells stimulated with ATP without fatty acid. EA: *p < 0.05; **p < 0.01; ***p < 0.001. LEA: ^##^p < 0.01; ^###^p < 0.001. TVA: ^†^p < 0.05 (vs ATP-stimulated cells without fatty acid).

Before assessing the toxicological properties of five major TFAs contained in foods (Fig. 1), we first examined whether a single treatment of each TFA has a cytotoxic effect on a mouse macrophage-like cell line, RAW264.7, and a microglial cell line, BV2. As shown in Fig. 2a and b, no significant change was observed in cell viability upon treatment of each TFA at 200 µM, indicating that only a single treatment of each TFA has no significant cytotoxic activity. Also, in line with our previous finding^11^, we confirmed that EA pretreatment strongly decreased cell viability upon eATP stimulation (Fig. 2c,d). We then undertook a toxicological evaluation of TFAs. RAW264.7 and BV2 cells were pretreated with various concentrations of TFAs, treated with 0.5 mM and 1.5 mM ATP, respectively, and assayed for cell viability. We found that pretreatment of iTFAs, including EA and LEA, strongly decreased cell viability, whereas that of rTFAs, including TVA, RA, and PEA, hardly did (Fig. 2e,f). To investigate the amount of TFAs incorporated into cells, we performed lipid analysis of RAW264.7 cells treated with each TFA at either 50 or 200 µM for 12 h using gas chromatography-tandem mass spectrometry (GC–MS/MS). Overall, iTFAs were more abundant in cells than rTFAs possibly due to more efficient incorporation or less metabolic rate; EA was the most abundant, while PEA was the least abundant and only about 1/5 of EA (Fig. S1a). Importantly, however, although the amount of incorporated rTFAs, namely TVA, RA, and PEA, in cells treated at 200 µM were equal to or more abundant than that of LEA in cells treated at 50 µM (Fig. S1a), rTFAs showed no significant pro-apoptotic effect at 50–200 µM as did LEA at 50 µM (Fig. 2c), indicating that rTFAs likely possess no or much less toxicity compared to iTFAs. Furthermore, we did not find any significant pro-apoptotic effect by pretreatment of either OA or LA, *cis* geometric isomers of EA and LEA, respectively (Fig. 2g,h). These data collectively suggest that among the tested UFAs, iTFAs have a unique pro-apoptotic role upon eATP treatment.

**Figure 3:**
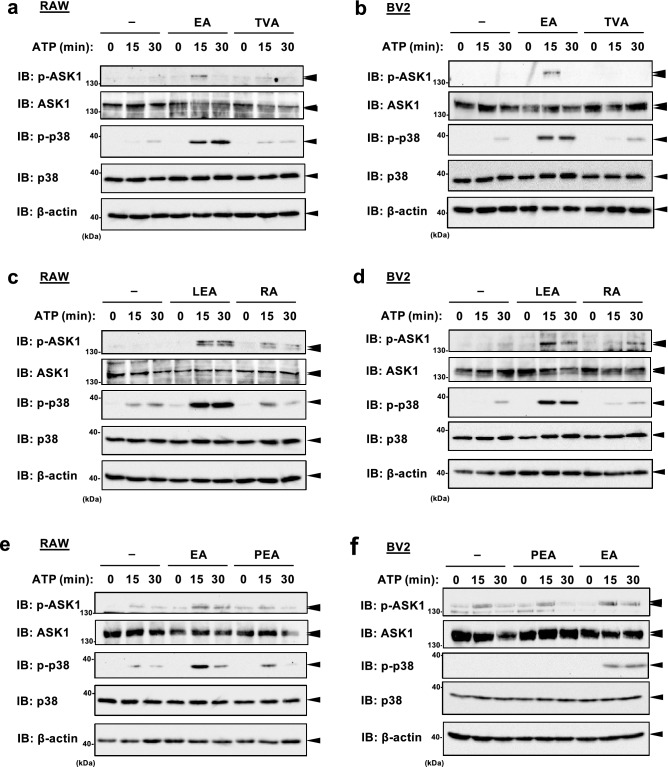
iTFAs specifically enhance activation of the ASK1-p38 MAP kinase pathway induced by eATP. (**a**–**f**) RAW264.7 (**a**–**c**) and BV2 (**d**–**f**) cells were pretreated with the indicated TFAs for 12 h at 200 µM, treated with ATP at 0.5 mM for 0, 15 or 30 min, and then subjected to immunoblotting with the indicated antibodies. Quantifications of immunoblots are shown in Supplementary Fig. S5. Original blots are presented in Supplementary Fig. S6.

We next evaluated the effect of TFAs on eATP-induced activation of the pro-apoptotic signaling, the ASK1-p38 MAP kinase pathway. Immunoblot analysis showed that pretreatment of either EA or LEA (iTFAs) apparently increased ATP-induced phosphorylation of ASK1 and p38 (Fig. 3a–d), while that of TVA or PEA did not (Fig. 3c–f), both in RAW264.7 and BV2 cells. Of note, pretreatment of RA slightly enhanced ATP-induced ASK1 phosphorylation both in RAW264.7 and BV2 cells for an unknown reason, but much more importantly, it hardly affected the phosphorylation status of its downstream MAP kinase p38 (Fig. 3c,d), which is more directly associated with the extent of apoptosis induced by eATP. Taken together, these results suggest that iTFAs specifically promote eATP-induced activation of the ASK1-p38 axis, and ultimately apoptosis.

### PUFAs suppress pro-apoptotic effect of EA

**Figure 4:**
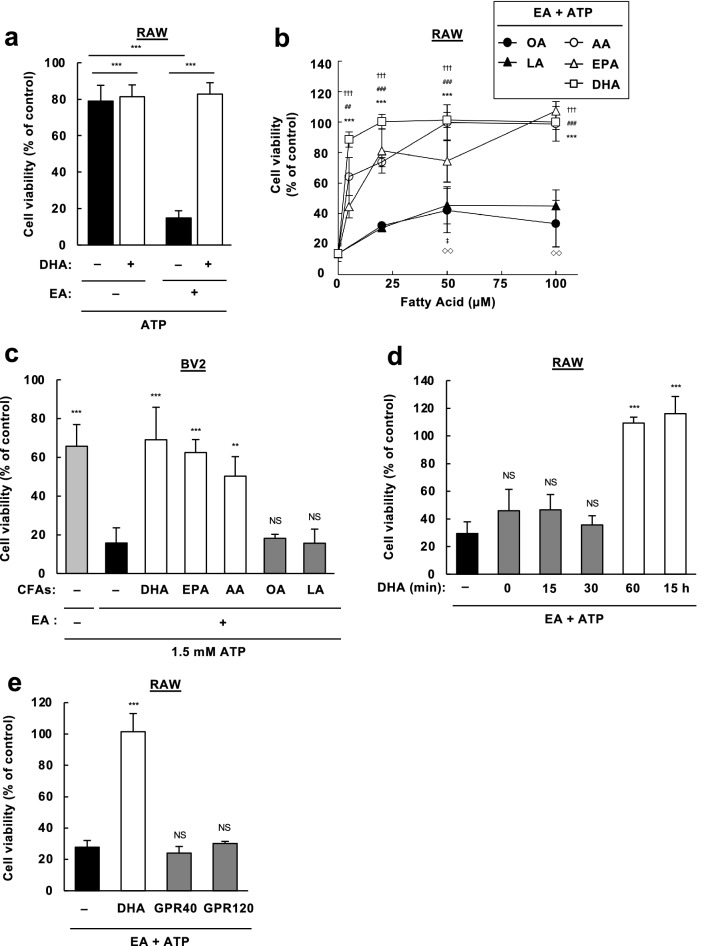
PUFAs strongly suppress pro-apoptotic action of EA during eATP stimulation. (**a**) RAW264.7 cells were pretreated with or without 200 µM EA and 50 µM DHA for 12 h, stimulated with 0.5 mM ATP for 6 h, and assayed for cell viability. Data shown are the mean ± SD (n = 3). ***p < 0.001. (**b**) RAW264.7 cells were treated with various concentrations of CFAs along with 200 mM EA for 12 h, stimulated with ATP at 0.5 mM for 6 h, and assayed for cell viability. Data are shown as relative cell viability (mean ± SD, n = 3), normalized with the viability of cells stimulated with ATP without any fatty acid. DHA: ***p < 0.001. EPA: ^##^p < 0.01; ^###^p < 0.001. AA: ^†††^p < 0.001. LA: ^◇◇^p < 0.01. OA: ^‡^p < 0.05 (vs ATP+, EA+, CFA–). (**c**) BV2 cells were pretreated with or without 200 µM EA and 50 µM DHA for 12 h, stimulated with 0.5 mM ATP for 6 h, and assayed for cell viability. Data are shown as relative cell viability (mean ± SD, n = 3), normalized with the viability of cells stimulated with ATP without any fatty acid. **p < 0.01; ***p < 0.001; NS, not significant (vs ATP+, EA+, CFA–). (**d**) RAW264.7 cells were pretreated with or without 200 µM EA for 15 h, and treated with 50 µM DHA for the indicated time periods before stimulation with 0.5 mM ATP for 6 h. Cell viability was assessed, and data are shown as relative cell viability (mean ± SD, n = 3), normalized with the viability of cells stimulated with ATP without any fatty acid. ***p < 0.001; NS, not significant (vs ATP+, EA+, DHA–). (**e**) RAW264.7 cells were pretreated with 200 µM EA in the presence of either 5 µM DHA, GPR40 ligand (TAK-875, 10 µM) or GPR120 ligand (TUG-891, 50 µM), stimulated with 0.5 mM ATP for 6 h, and then assayed for cell viability. Data are shown as relative cell viability (mean ± SD, n = 3), normalized with the viability of cells stimulated with ATP without any fatty acid or ligand. ***p < 0.001; NS, not significant (vs ATP+, EA+, DHA or inhibitor–).

DHA is a representative PUFA that is well-known for its protective role in diverse diseases, including CVDs and NDs^17^. We therefore examined whether DHA is protective against EA toxicity, and surprisingly, found that co-pretreatment of DHA almost completely reversed EA-dependent decrease in cell viability upon eATP stimulation (Fig. 4a). To compare the capacity of this inhibitory effect among CFAs, we pretreated various concentrations of CFAs with EA and observed cell viability upon stimulation with eATP. As shown in Fig. 4b and 4c, whereas co-pretreatment of OA or LA only marginally affected cell viability, that of either arachidonic acid (AA, C20:4 *c*5,*c*8,*c*11,*c*14), EPA or DHA drastically increased cell viability, suggesting that PUFAs, particularly those possessing four or more double bonds, have a strong protective effect against EA toxicity upon eATP stimulation. Since DHA had the highest inhibitory effect among PUFAs (Fig. 4b), hereafter we mainly used DHA for further analysis. Before further analysis, we first checked whether DHA affects the amount of EA incorporated into cells. GC–MS/MS analyses showed that DHA co-treatment did not reduce the amount of EA in RAW264.7 cells (Fig. S1b), while a substantial amount of DHA was incorporated in a concentration-dependent manner (Fig. S1c). We also confirmed that co-treatment of EA did not prevent the incorporation of DHA (Fig. S1c). Furthermore, a single treatment of DHA did not affect cell viability in both RAW264.7 and BV2 cells (Fig. S2). DHA exists as a variety of forms, such as free fatty acids, and acyl moieties of triacylglycerols (TAGs) and phospholipids, and acts in multiple ways as a ligand, a precursor of metabolic products, and a component of membrane lipids^17^. To determine the mode of action of DHA, we first investigated how much time is required for the pretreatment of DHA to exert its protective effect on EA toxicity. Cell viability assay showed that DHA pretreatment within 30 min did not reverse the pro-apoptotic effect of EA, while that for 60 min or 15 h completely reversed it (Fig. 4d), implicating that to protect cells from EA toxicity, exogenously added DHA may not serve as a ligand for receptors located on the plasma membrane, and may require cellular incorporation and metabolic processes which takes about 1 h. In line with this notion, pretreatment of TAK-875 and TUG-891, synthetic ligands for G protein-coupled receptor 40 (GPR40) and GPR120, respectively, which are known as PUFA receptors^18^, did not inhibit EA-mediated increase in cell death as did DHA pretreatment (Fig. 4d). To identify the metabolic process that is associated with the protective role of DHA, we tested a wide variety of chemical inhibitors for each process of DHA metabolism: Aspirin, Celecoxib (cyclooxygenase (COX) inhibitors), Zileuton, ML355, PD146176 (lipoxygenase (LOX) inhibitors), HET0016, 1-ABT, MS-PPOH (cytochrome P450 (CYP450) inhibitors), and Triacsin C (a long-chain acyl-CoA synthetase (ACSL) inhibitor). However, none of these inhibitors canceled the protective effect of DHA on EA toxicity (Fig. S3). Collectively, these data suggest that DHA strongly suppresses the pro-apoptotic action of EA in response to eATP possibly in an unconventional manner that is not related to fatty acid receptors and the typical metabolic process of DHA.

### DHA prevents CaMKII-dependent hyperactivation of ASK1

**Figure 5:**
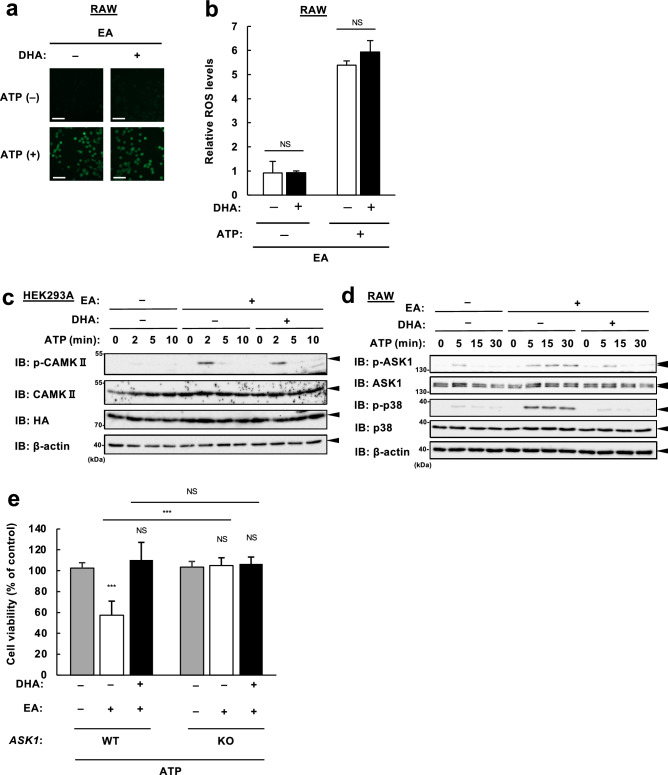
DHA prevents EA-dependent hyperactivation of the ASK1-p38 MAP kinase pathway. (**a**, **b**) RAW264.7 cells were pretreated with or without 200 µM EA and 50 µM DHA for 12 h. After incubation with 10 µM DCFH-DA for 30 min, cells were stimulated with 0.5 mM ATP for 5 min, and intracellular ROS was visualized by a confocal microscopy (**a**). ROS levels were measured and shown as mean ± SD (n = 3) (**b**). Scale bar, 100 µm. NS, not significant. (**c**) HEK293A cells were transfected with CaMKII and HA-P2X_7_ for 12 h, pretreated with the indicated fatty acids for 12 h, and stimulated with 3 mM ATP for the indicated times. Cell lysates were subjected to immunoblotting with the indicated antibodies. (**d**) RAW264.7 cells were pretreated with or without 200 µM EA and 50 µM DHA for 12 h, treated with 0.5 mM ATP for the indicated time periods, and then subjected to immunoblotting with the indicated antibodies. (**e**) Wild-type (WT) and *ASK1* knockout (KO) RAW264.7 cells were pretreated with or without 200 µM EA and 50 µM DHA for 12 h, stimulated with 0.5 mM ATP for 6 h, and assayed for cell viability. Data shown are the mean ± SD (n = 3). ***p < 0.001; NS, not significant. Quantifications of immunoblots are shown in Supplementary Fig. S5. Original blots are presented in Supplementary Fig. S6.

We previously demonstrated that ROS produced by NADPH oxidase and Ca^2+^/calmodulin-dependent protein kinase II (CaMKII), an upstream kinase of ASK1, are essential for EA-mediated hyperactivation of ASK1 in response to eATP^11^. To determine the target of DHA to exert its protective action against EA toxicity, we examined the effect of DHA on eATP-induced ROS generation and CaMKII phosphorylation. Using a green fluorescent ROS indicator, DCFH-DA, we observed no significant difference in eATP-induced ROS generation with or without DHA pretreatment (Fig. 5a,b). Furthermore, immunoblot analysis showed that DHA did not affect EA-dependent hyperphosphorylation of CaMKII at Thr-286 (Fig. 5c), but clearly suppressed that of ASK1 and p38 (Fig. 5d). These data suggest that DHA targets ASK1 and thereby blocks pro-apoptotic signaling amplified by EA. To further consolidate this finding, we examined the anti-apoptotic effects of DHA in ASK1 knockout (KO) RAW264.7 cells established in our previous study^11^. As shown in Fig. 5e, whereas EA promoted eATP-induced cell death and DHA reversed it in wild-type (WT) cells, neither pro-apoptotic effect of EA nor anti-apoptotic effect of DHA was observed in ASK1 KO cells, supporting the notion that DHA exerts a protective role in EA-mediated enhancement of eATP-induced apoptosis by targeting ASK1.

## Discussion

**Figure 6:**
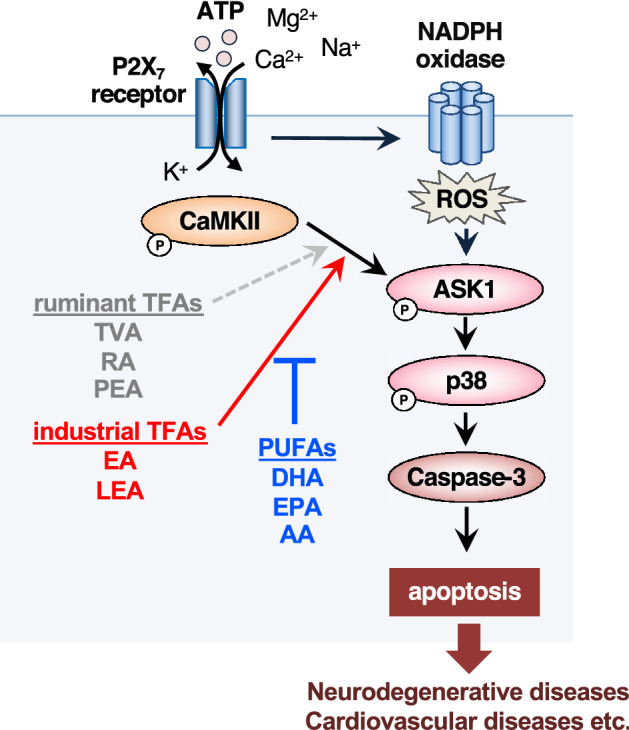
Schematic illustration of the toxic actions of TFAs and the protective actions of PUFAs. Upon eATP stimulation, iTFAs, such as EA and LEA, potently enhanced CaMKII-dependent ASK1 activation and apoptosis (solid red arrow), whereas rTFAs, such as TVA, RA, and PEA, did not (dotted gray arrow). PUFAs, such as DHA, EPA, and AA, strongly blocked pro-apoptotic effect of EA possibly by targeting ASK1, which could be utilized for preventing and combating TFA-related diseases.

In this study, we demonstrated that EA and LEA, the predominant TFAs in industrially produced foods (iTFAs), but not TVA, RA, and PEA, major TFAs contained in ruminant foods (rTFAs), efficiently facilitated eATP-induced apoptosis signaling (Figs. 2e,f and 3). Pro-apoptotic action of iTFAs was not observed from OA and LA, geometric *cis* isomers of EA and LEA, respectively (Figs. 2g,h and 3). Collectively, these results suggest that iTFAs possess a unique ability to promote eATP-induced apoptosis, which can well account for their particular epidemiological link with TFA-related diseases, such as CVDs and NDs^19,20^ (Fig. 6). Based on the difference in the positions of carbon–carbon double bonds between iTFAs and rTFAs, it can be speculated that n-9 *trans* double bond common between EA and LEA is necessary for their pro-apoptotic action. While EA enhances eATP-induced apoptosis to the same extent as LEA (Fig. 2e,f), mono-ene TFAs (ex. EA) are generally more abundant than di-ene TFAs in diet (ex. LEA)^21^; consistently, the plasma level of EA in human (~ 10 µM) is much higher than that of LEA (~ 2 µM)^22^. Therefore, EA likely plays a major causative role in TFA-related diseases among TFA species. Notably, although there is no reliable evidence of the actual TFA amount in human tissues, EA concentration in the liver of rodents fed with a normal diet is estimated to be 1–6 mM^10,11,23,24^, and that in rat brain is estimated to be 200 µM^25^. Therefore, the concentration of TFAs at which significant toxicity was observed (50–200 µM) in this study would be physiologically relevant.

We also demonstrated that PUFAs, such as AA, EPA, and DHA, possess a strong protective effect on TFA toxicity. Among these PUFAs, DHA was most effective in suppressing the pro-apoptotic action of EA, and only 5 µM of DHA was enough to reverse its toxicity (Fig. 4b). Mechanistically, DHA prevented hyperactivation of ASK1 and p38 by EA (Fig. 5d), but not that of CaMKII (Fig. 5c), suggesting that the direct target of PUFAs is ASK1. The protective action of DHA is not likely mediated by either fatty acid receptors, GPR40 and GPR120 (Fig. 4e), incorporation into phospholipids, or enzymatic metabolization by lipoxygenases or cytochrome P450 (Fig. S3), and only one hour of treatment was enough for DHA to exert its protective effect. (Fig. 4d). Taken together, it can be assumed that DHA suppresses EA-mediated ASK1 hyperactivation most likely as a non-esterified free fatty acid. Intriguingly, protein phosphatase 5 (PP5), a negative regulator of ASK1 phosphorylation, is known to be activated by direct interaction with PUFAs such as AA^26^. Thus, it would be possible that PUFAs directly bind to PP5, elevate the phosphatase activity of PP5, and thereby prevent ASK1 hyperactivation in response to EA. Nevertheless, it is noteworthy that while DHA completely reversed the pro-apoptotic effect of EA, it did not increase cell viability when stimulated with eATP without EA (Fig. 4a,c), implying that PUFAs preferentially suppress EA-dependent hyperactivation of ASK1, rather than normal activation of ASK1 upon eATP stimulation without EA. In line with this notion, DHA did not inhibit ASK1/p38 activation upon treatment of lipopolysaccharide (LPS) or H_2_O_2_, strong activators of the ASK1-p38 MAP kinase pathway (Fig. S4). Given that the most prominent target of EA is CaMKII, another possibility is that DHA diminishes CaMKII-dependent phosphorylation of ASK1 by interfering with their interaction through an unknown mechanism, thereby preventing hyperactivation of ASK1. These possibilities should be tested in future studies to clarify the underlying molecular mechanism.

Accumulating evidence has shown that consumption of PUFAs, including EPA and DHA, decreases the risk of various diseases such as CVDs and NDs, which has been largely attributed to their anti-inflammatory functions^27,28^. Clinical trials have been performed to examine the potential effects of EPA/DHA supplementation on these diseases, and several of them revealed its benefit in reducing CV events and mortality, and improving Parkinson’s disease and Alzheimer’s disease^28,29^. However, to the best of our knowledge, no previous epidemiological or clinical study has investigated whether PUFA intake could alleviate diseases associated with TFA intake. Intriguingly, several lines of evidence have demonstrated an inverse relationship between plasma concentrations of TFAs and PUFAs^30–32^. A similar inverse relationship was also observed in the plasma and liver of rats fed with a TFA-containing diet, which was considered to be due to the low activity of delta-6 fatty acid desaturase in their liver that is necessary for PUFA synthesis^25^. Taking into account the protective role of PUFAs in TFA toxicity, TFA consumption contributes to disease pathogenesis and progression possibly by two mechanisms: directly by promoting cell death and inflammation, and indirectly by decreasing the amount of PUFAs. PUFA supplementation could be utilized as a promising therapeutic approach to prevent and mitigate TFA-related diseases, such as CVDs and NDs. As we recently revealed other toxicity mechanisms of TFAs triggered by DNA damage^33,34^, we would like to conduct a similar comprehensive toxicity assessment of TFAs in a future study to fully understand the difference in toxicity of TFAs in the case of DNA damage, and to explore the potential application of PUFAs in the treatment of their toxicity.

## Methods

### Reagents

ATP and H_2_O_2_ was purchased from Wako (Tokyo, Japan). LPS was purchased from Invivogen (San Diego, CA, USA). Aspirin was purchased from Tokyo Kasei (Tokyo, Japan). 1-Aminobenzotriazole, HET0016, Zileuton, PD146176, Celecoxib, MS-PPOH, ML355, and Triacsin C were purchased from Cayman (Ann Arbor, MI, USA).

### Cell culture

RAW264.7 and BV2 cells, obtained from ATCC, were cultured in RPMI 1640 and Dulbecco’s Modified Eagle Medium (Nakalai Tesque, Kyoto, Japan), respectively, containing 10% heat-inactivated fetal bovine serum (Sigma, Burlington, MA, USA) and 1% penicillin–streptomycin solution (Nakalai Tesque) in 5% CO_2_ at 37 °C. ASK1 KO RAW264.7 cells were established in our previous study^11^. HEK293A cells obtained from Thermo Fisher Scientific (Waltham, MA, USA) were cultured in Dulbecco’s Modified Eagle Medium (Nakalai Tesque) containing 5% heat-inactivated fetal bovine serum (Sigma, Burlington, MA, USA) and 1% penicillin–streptomycin solution (Nakalai Tesque) in 5% CO_2_ at 37 °C.

### Plasmids and trasnfection

pcDNA3.2 HA-mouse P2X_7_ and pCAGGS-rat CaMKIIα were constructed in previous studies^11,35^. Plasmid transfection was performed using Polyethylenimine “Max” (PEI-MAX, Polysciences), according to the manufacturer’s instructions.

### Preparation and treatment of fatty acids

OA (Nakalai Tesque), EA (Sigma), LA, LEA, AA, EPA, DHA (Cayman), TVA (Olbracht Serdary Research Laboratories, Toronto, ON, Canada), and RA and PEA (Funakoshi, Tokyo, Japan) were prepared as described previously^33^. Briefly, fatty acids were dissolved in 0.1 N NaOH at 70 °C, and then conjugated with fatty acid-free BSA (Wako, pH 7.4) at 55 °C for 10 min to make 5 mM BSA-conjugated fatty acid stock solutions containing 10% BSA. Cells were treated with various concentrations of BSA-conjugated fatty acids by diluting stock solutions in a medium without fetal bovine serum (final BSA concentration was set to 1%).

### Immunoblot analysis

Cells were lysed in ice-cold lysis buffer containing 20 mM Tris–HCl, pH 7.4, 150 mM NaCl, 1% Triton-X100, 10% Glycerol, and 1% protease and phosphatase inhibitor cocktail (Nacalai tesque). After centrifugation, the cell extracts were resolved by SDS-PAGE, and were analyzed as described previously^36^. The antibodies used for immunoblotting were against phospho-ASK1 (Thr-845), phospho-p38, p38 (Cell Signaling), β-actin (Santa cruz, Dallas, TX, USA), ASK1 (Wako and Abcam, Cambridge, UK), phospho-CaMKII (Thr-286), and CaMKII (gift from Dr. Kohji Fukunaga, Tohoku University)^37^. The blots were developed with ECL (Merck Millipore, Burlington, MA, USA), and detected with ChemiDoc Touch Imaging System (BioRad, Hercules, CA, USA).

### Cell viability assay

Cell viability was assayed as described previously^38^. RAW264.7 and BV2 cells were seeded on 96-well plates. After any stimulation or treatment, cell viability was determined using Cell Titer 96 Cell Proliferation Assay (Promega, Madison, WI, USA), according to the manufacturer’s protocol. The absorbance was read at 490 nm using a microplate reader (iMark microplate reader, Biorad). Data are normalized to control without stimulus, unless noted otherwise.

### Bioimaging and quantification of intracellular ROS

Intracellular ROS was detected and quantified as described previously^39^. Briefly, after stimulation, cells seeded on a glass plate were treated with 10 μM DCFH-DA (Sigma) for 30 min, and intracellular ROS generation was observed using a Zeiss LSM800 laser confocal microscope (Carl Zeiss, Oberkochen, Germany) and the images were processed with Zen software. After background subtraction, ROS level was calculated by dividing total green fluorescence by cell numbers using Image J. Data are shown as mean ± SD of relative fluorescence intensity from three different fields of view.

### Lipid analysis

Lipids were extracted by the method of Bligh and Dyer^40^. Isolated lipids were methylated with 2.5% H_2_SO_4_ in methanol. The resulting fatty acid methyl esters were then extracted with hexane and subjected to gas chromatography tandem mass spectrometry (GC–MS/MS) analysis. GC–MS/MS analysis was performed by using a GCMS-QP2010 Plus (Shimadzu, Kyoto, Japan) equipped with Zebron ZB-FAME 60 m × 0.25 mm × 0.20 µm (Phenomenex, Torrance, CA, USA). The oven temperature program was as follows: the initial temperature was 100 °C for 2 min, then raised to 280 °C at 15 °C/min, and held for 5 min. The injector and detector temperatures were set at 240 °C and 260 °C, respectively. The amounts of incorporated TFAs were calculated based on standard curves created from serial dilution of the respective fatty acids, which was normalized with the amount of extracted protein measured by Bradford method using protein assay CBB solution (Nakalai Tesque).

### Statistics

All the values are expressed as means ± SD, and statistical analyses were performed using GraphPad Prism software (v.9.3.0). All experiments were repeated at least three independent times. Two groups were compared using two-tailed Student’s t-test. Multiple-group comparisons were conducted using either the one-way ANOVA analysis or two-way ANOVA analysis followed by Tukey–Kramer or Dunnett’s test.

## Supplementary Information

Supplementary Figures.

## Data Availability

The datasets used and/or analyzed during the current study available from the corresponding author on reasonable request.
