# Supplementary material for: A comprehensive toxicological analysis of trans-fatty acids (TFAs) reveals a pro-apoptotic action specific to industrial TFAs counteracted by polyunsaturated FAs
Source: Sci Rep. 2023 Apr 11;13:5883. doi: 10.1038/s41598-023-32083-9 (PMC10090069; doi:10.1038/s41598-023-32083-9)
Supplement: Supplementary file 1 — Supplementary Figures. [file 41598_2023_32083_MOESM1_ESM.pdf]

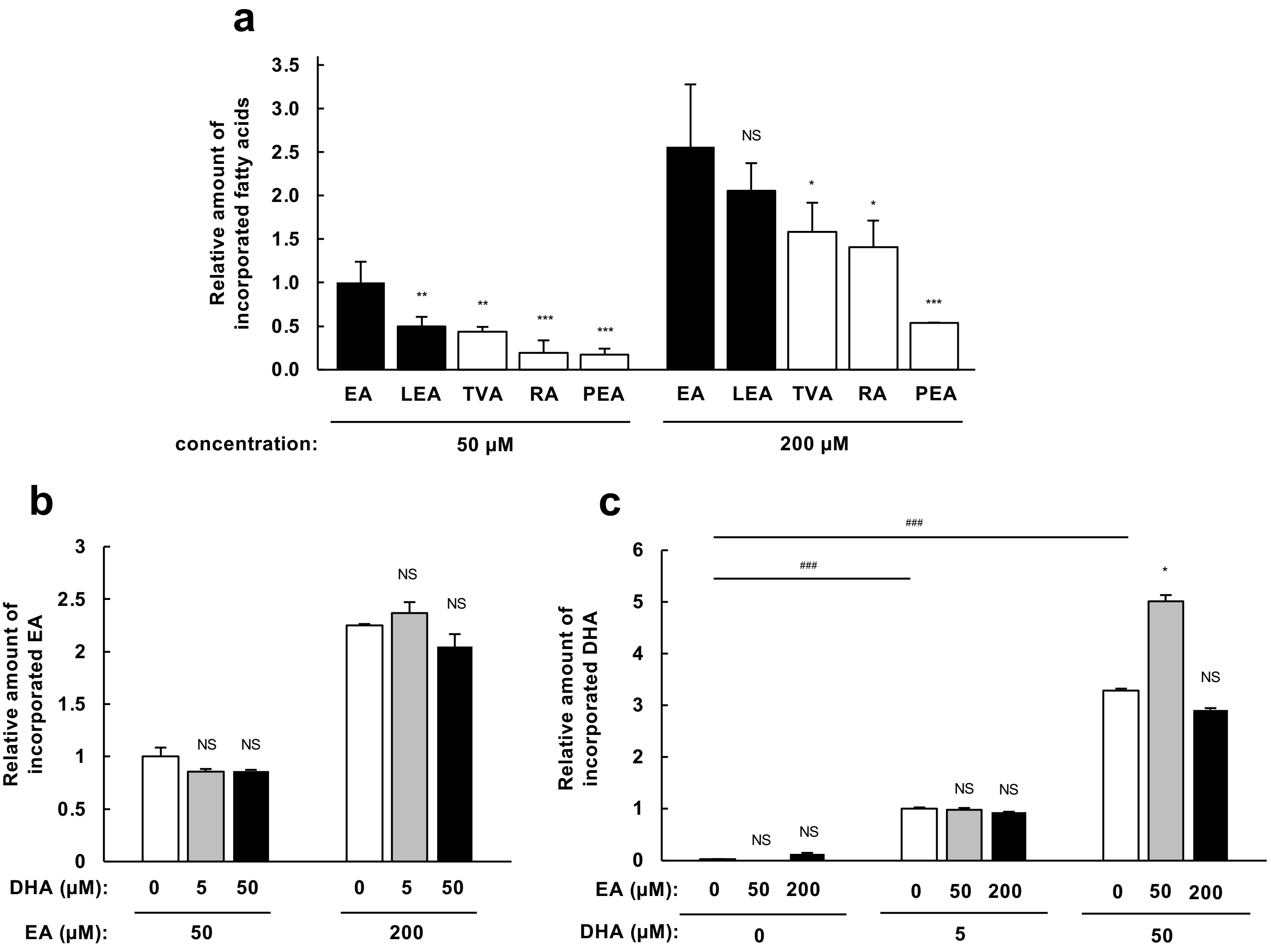

Supplementary Figure 1. Comparison of the amounts of fatty acids incorporated into cells
a-c) RAW264.7 cells were treated with the indicated TFAs and/or DHA for 12 h. Lipids were extracted from cells, derivatized by methylation, and then subjected to GC-MS analysis. Relative molar amounts of incorporated TFAs (a), EA (b), and DHA (c) are shown as mean ± SD (n=3). *,p<0.05; **,p<0.01; ***,p<0.001; NS, not significant (vs 50 or 200 μM EA, a and b; vs 0, 5, or 50 μM DHA, c); $^{###}$,p<0.001.

Hirata et al., Fig. S1.

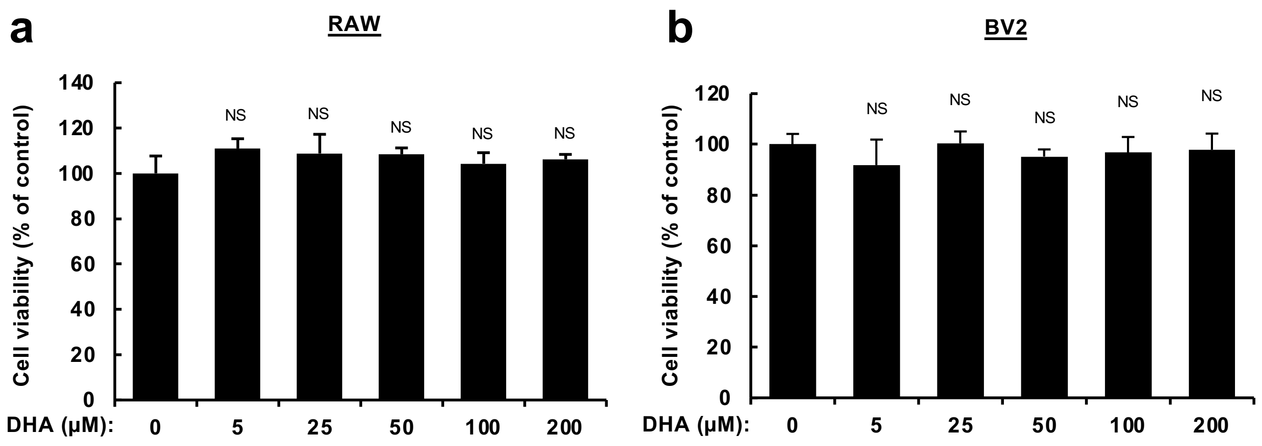

Supplementary Figure 2. DHA does not affect cell viability

RAW264.7 (a) and BV2 (b) cells were treated with DHA at the indicated concentrations for 12 h, and assayed for cell viability. Data are shown as mean ± SD (n=3). NS: not significant (vs control without DHA).

Hirata et al., Fig. S2.

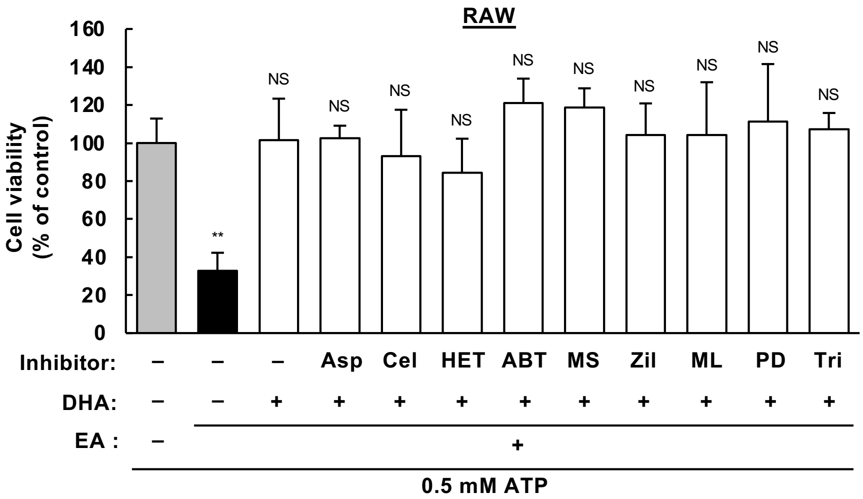

Supplementary Figure 3. DHA metabolic pathway inhibitors do not block its protective effect on EA toxicity

RAW264.7 cells were pretreated with or without 200 μM EA for 12 h, treated with the indicated inhibitors for 30 min followed by treatment with 50 μM DHA for 1 h, and stimulated with 0.5 mM ATP for 6 h. Data are shown as relative cell viability (mean ± SD, n=3), normalized with the viability of cells stimulated with ATP without any fatty acid or inhibitor. Asp: Aspirin (COX inhibitor), 500 μM; Cel: Celecoxib (COX2 selective inhibitor), 10 μM; HET: HET0016 (selective CYP inhibitor: CYP2C9/CYP2D6/CYP3A4); ABT: 1-Aminobenzotriazole (non-specific CYP inhibitor), 5 μM; MS: MS-PPOH (selective CYP450 epoxidase inhibitor), 20 μM; Zil: Zileuton (5-LOX inhibitor), 0.5 μM; ML: ML335 (12-LOX inhibitor), 10 μM; PD: PD146176 (15-LOX inhibitor), 5 μM; Tri: Triacsin C (ACSL inhibitor), 10 μM. **,p<0.01; NS, not significant (vs ATP+, EA–, DHA–, inhibitor–).

Hirata et al., Fig. S3.

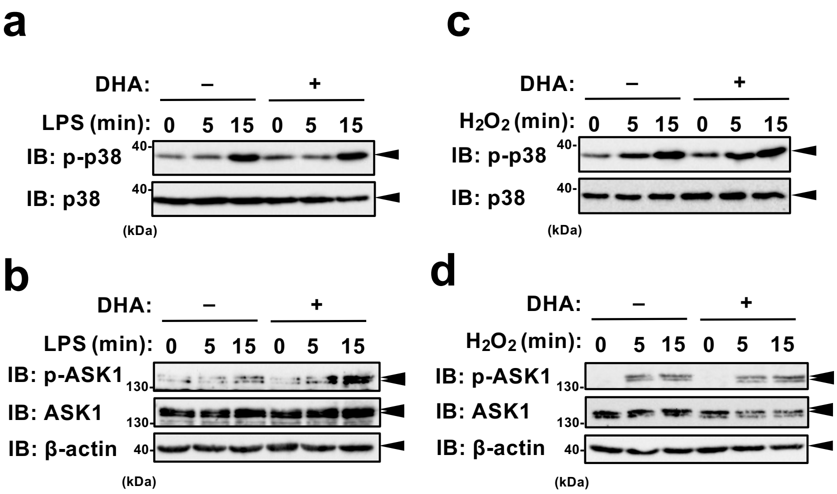

Supplementary Figure 4. DHA does not affect ASK1-dependent p38 activation
(a-d) RAW264.7 cells were pretreated with 50 μM DHA for 12 h, treated with 1 μg/ml LPS (a, b) or 1 mM H_2O_2 (c, d) for the indicated time periods, and then subjected to immunoblotting with the indicated antibodies.

Hirata et al., Fig. S4.

a Fig. 3a

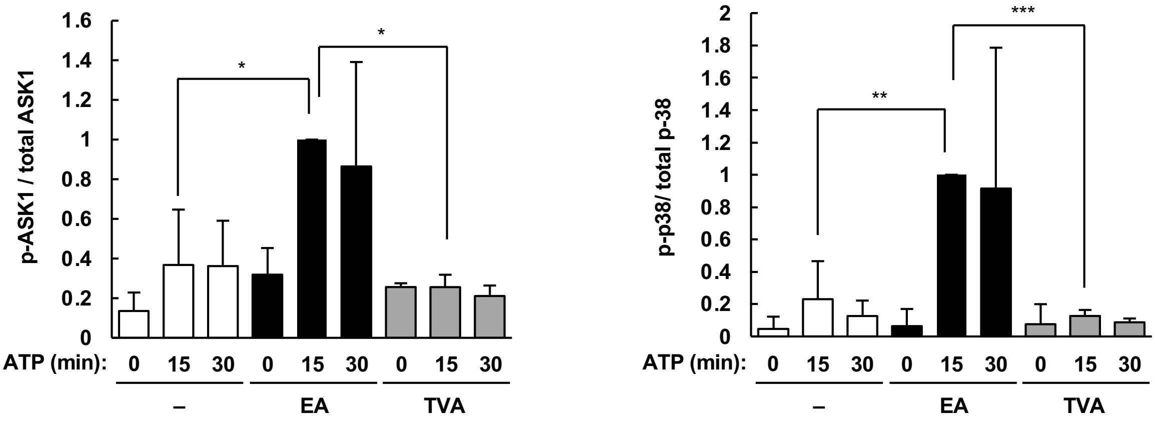

b Fig. 3b

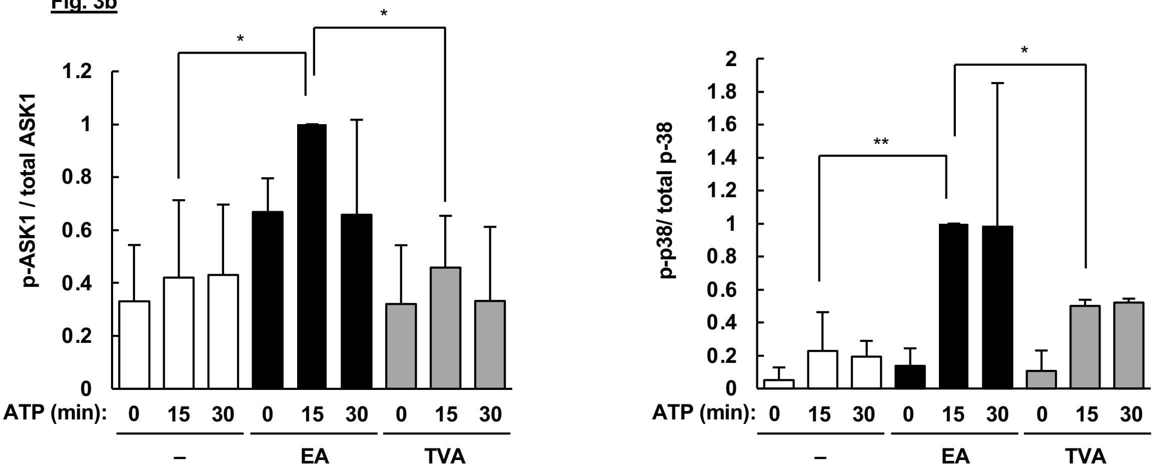

c Fig. 3c

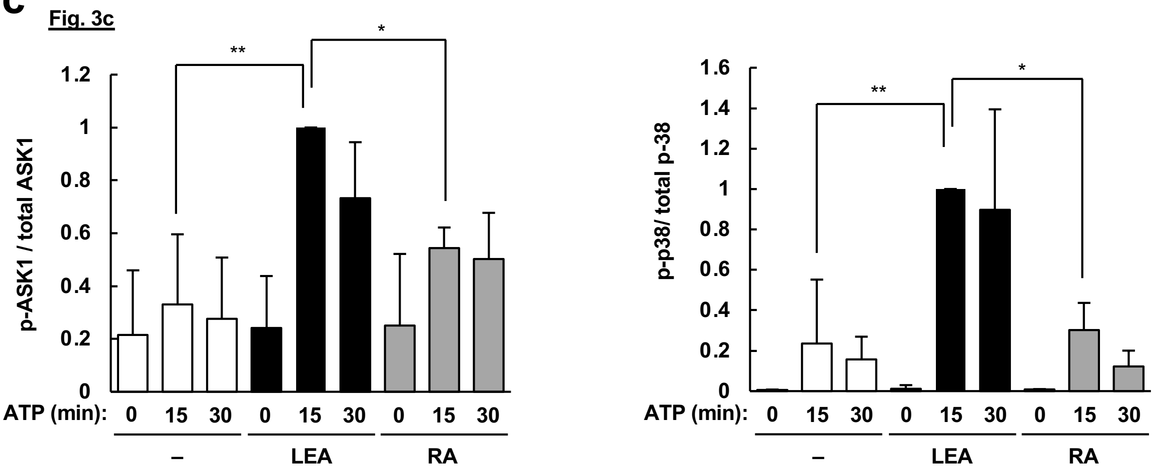

Hirata et al., Fig. S5.

d Fig. 3d

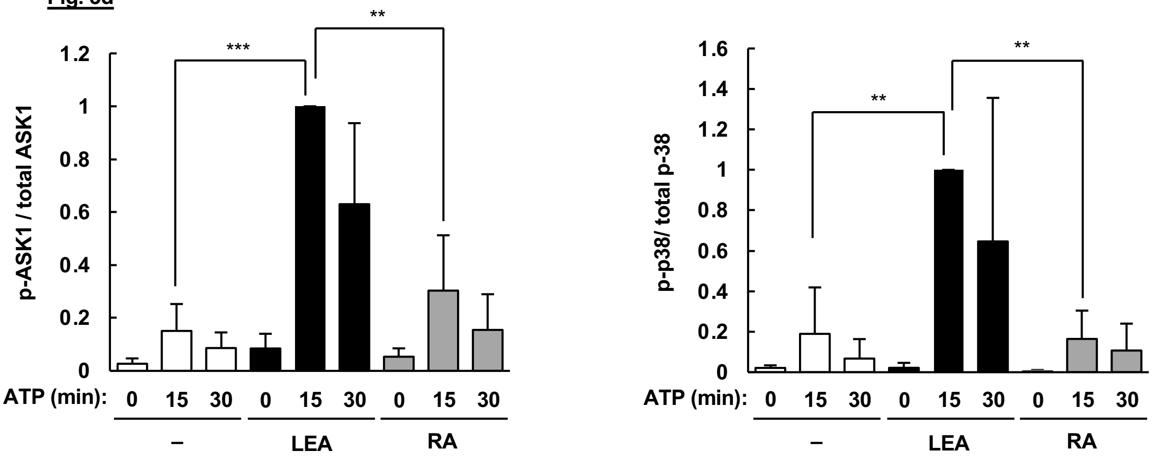

e Fig. 3e

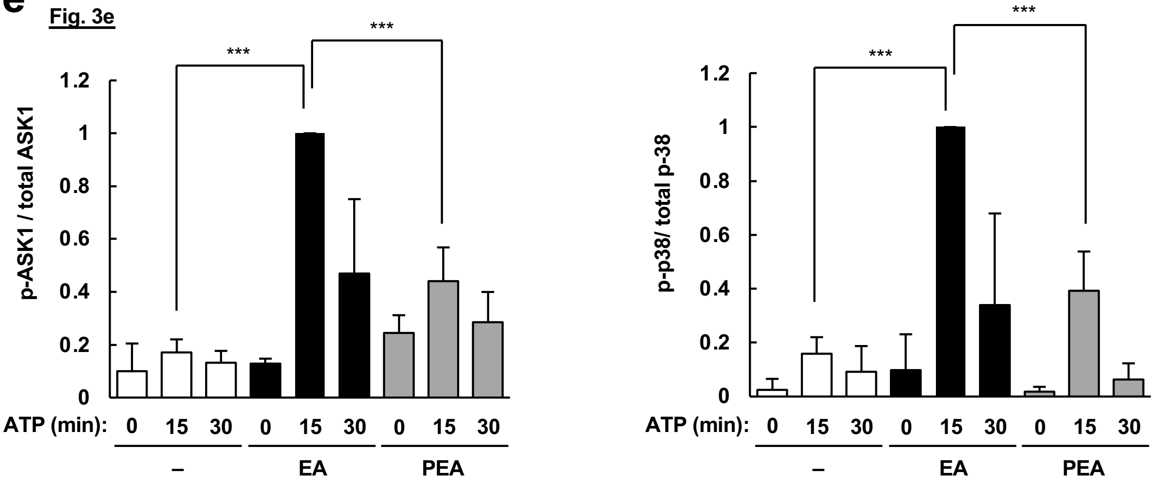

f Fig. 3f

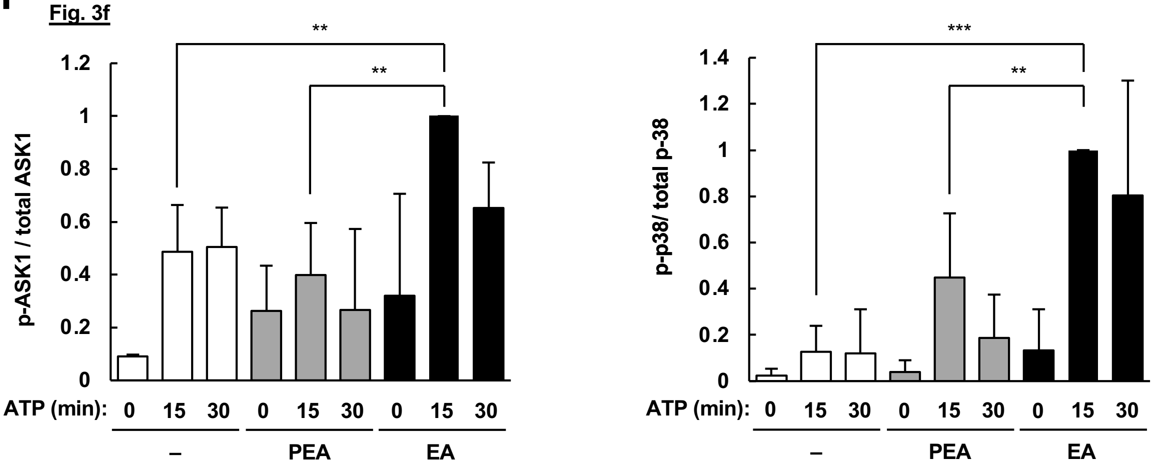

Hirata et al., Fig. S5.

g Fig. 5c

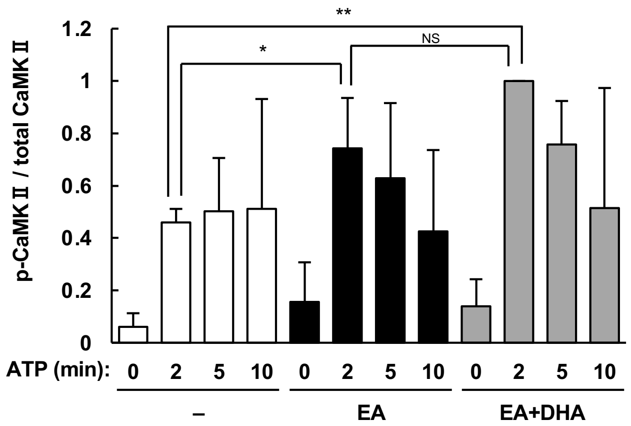

h Fig. 5d

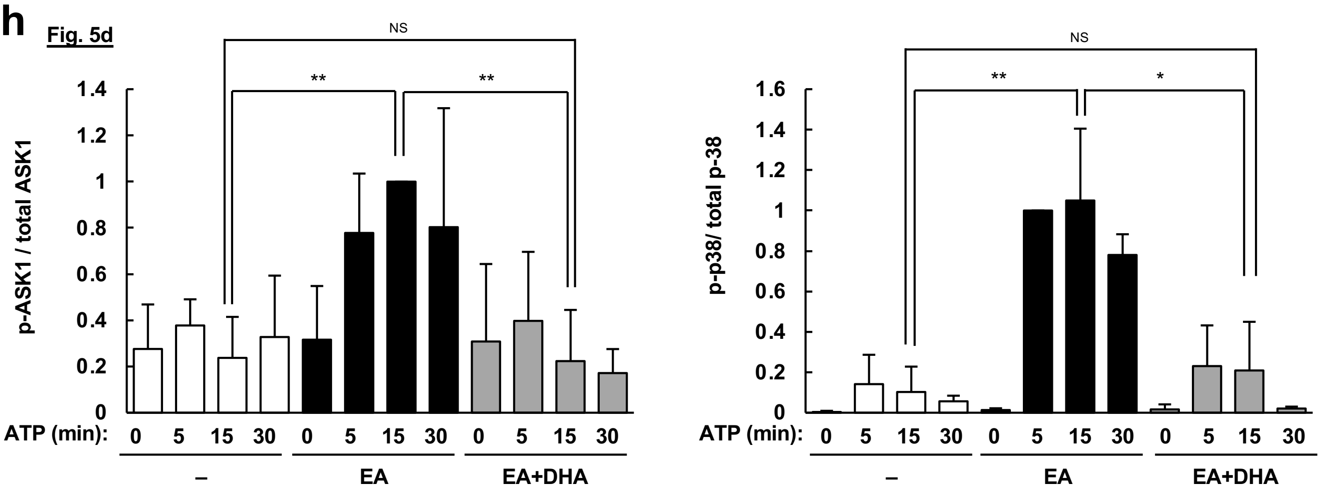

Supplementary Figure 5. Quantification of immunoblotting data

(a-h) Quantified relative band intensity of immunoblot data are shown as mean ± SD (f, n=4; others, n=3): Fig. 3a (a), Fig. 3b (b), Fig. 3c (c), Fig. 3d (d), Fig. 3e (e), Fig.3f (f), Fig. 5c (g), Fig. 5d (h).

Hirata et al., Fig. S5.

a

<u>Fig. 3a</u>

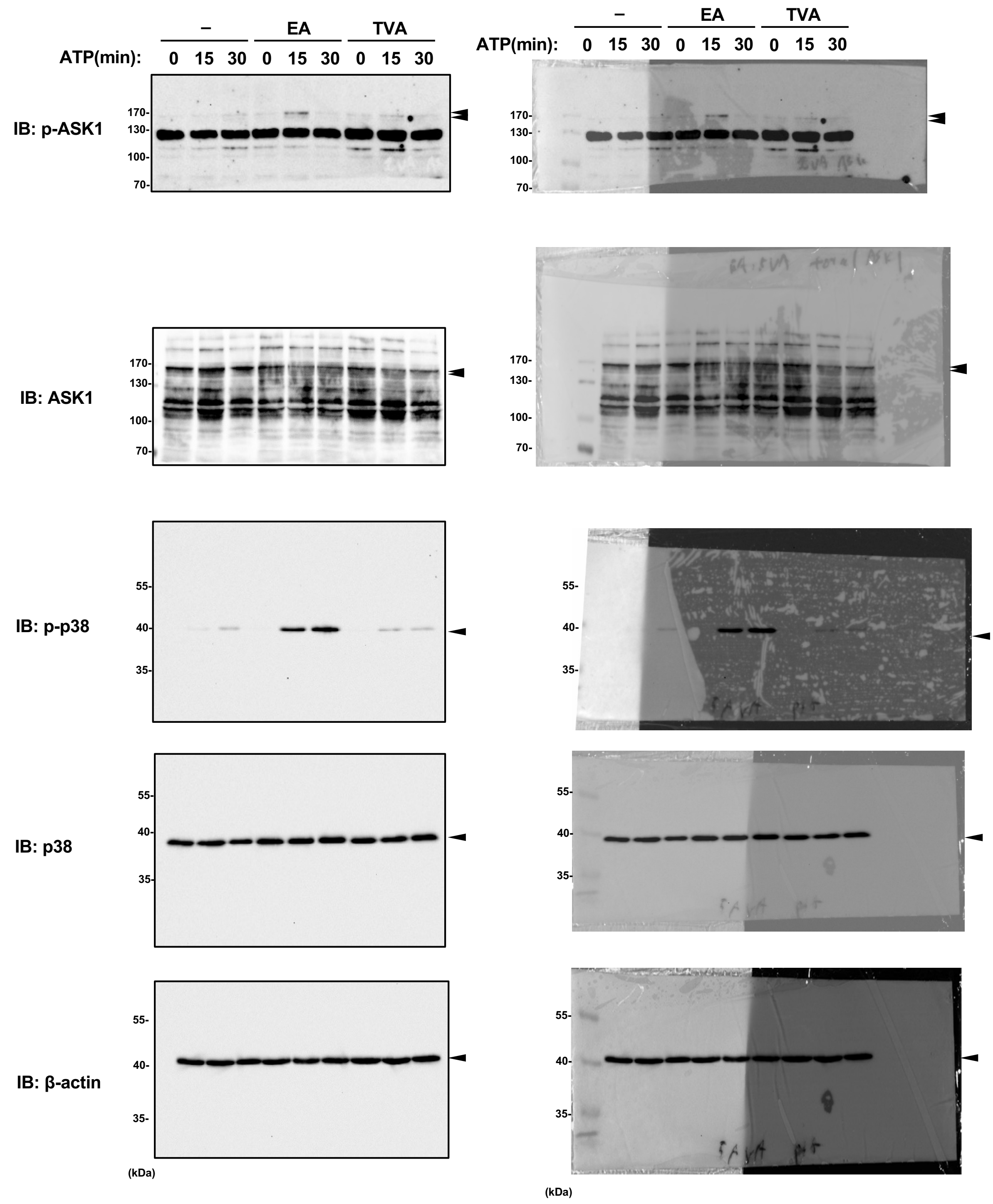

Hirata et al., Fig. S6.

b

Fig. 3b

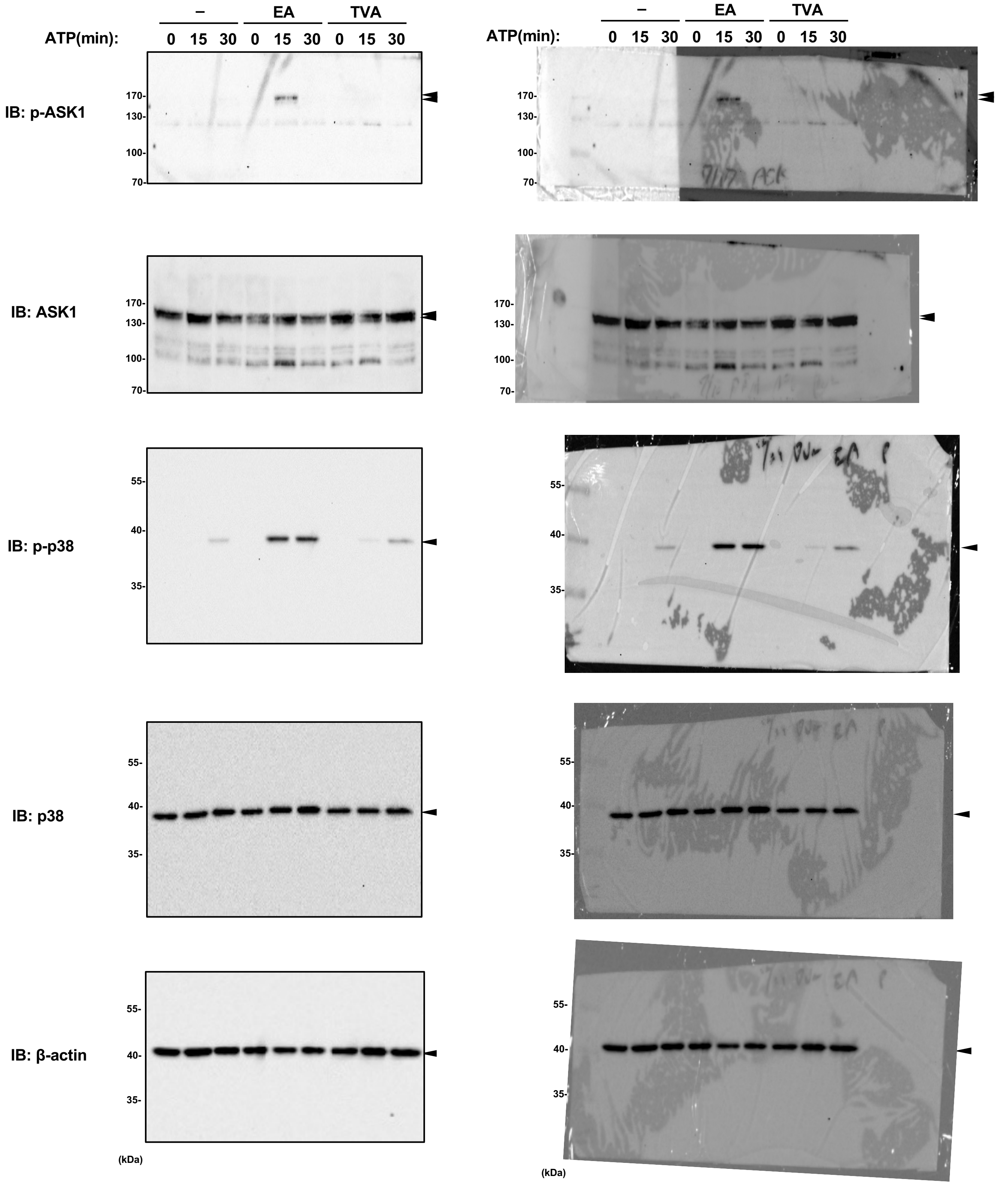

Hirata et al., Fig. S6.

c

Fig. 3c

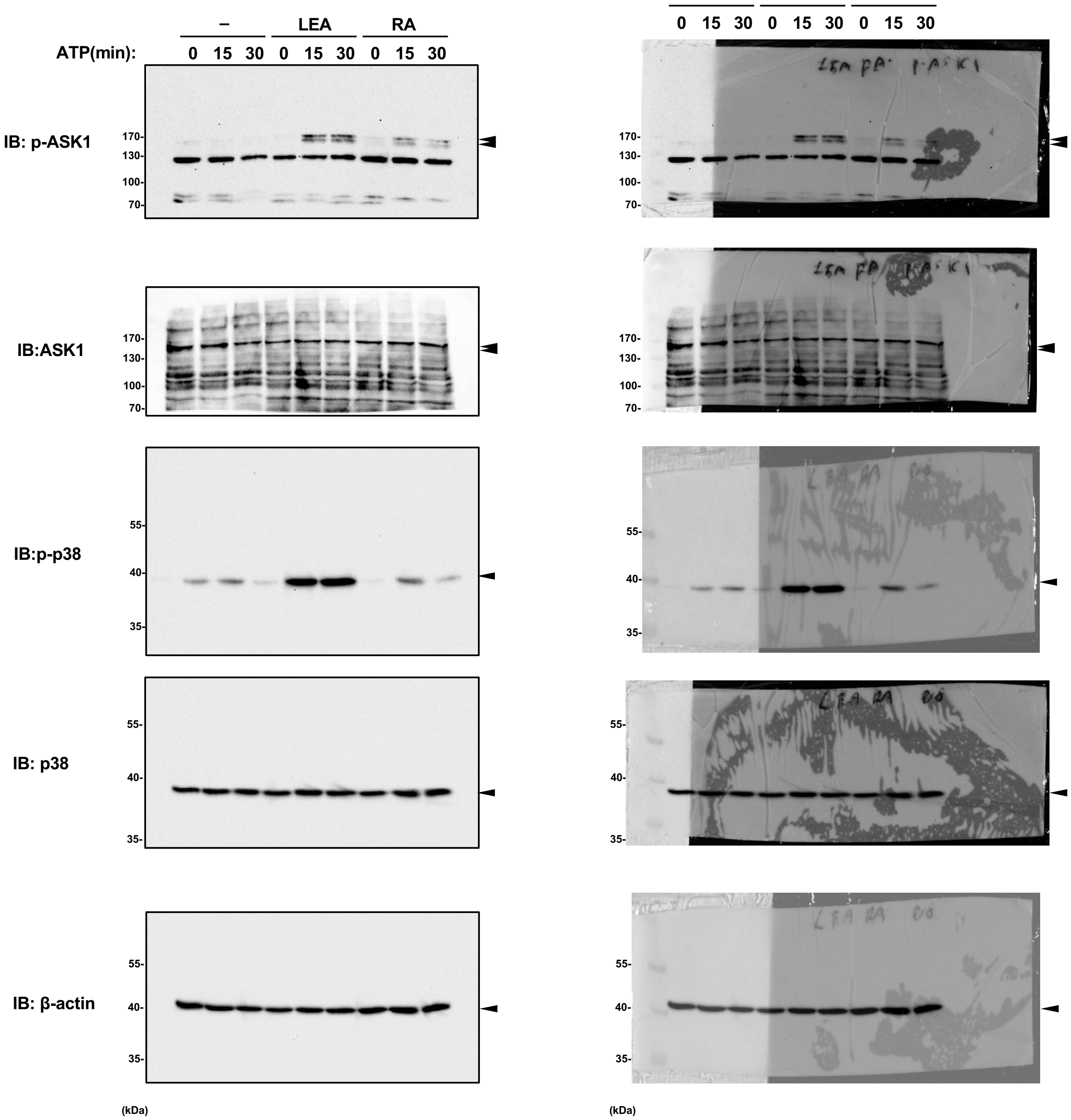

Hirata et al., Fig. S6.

d

Fig. 3d

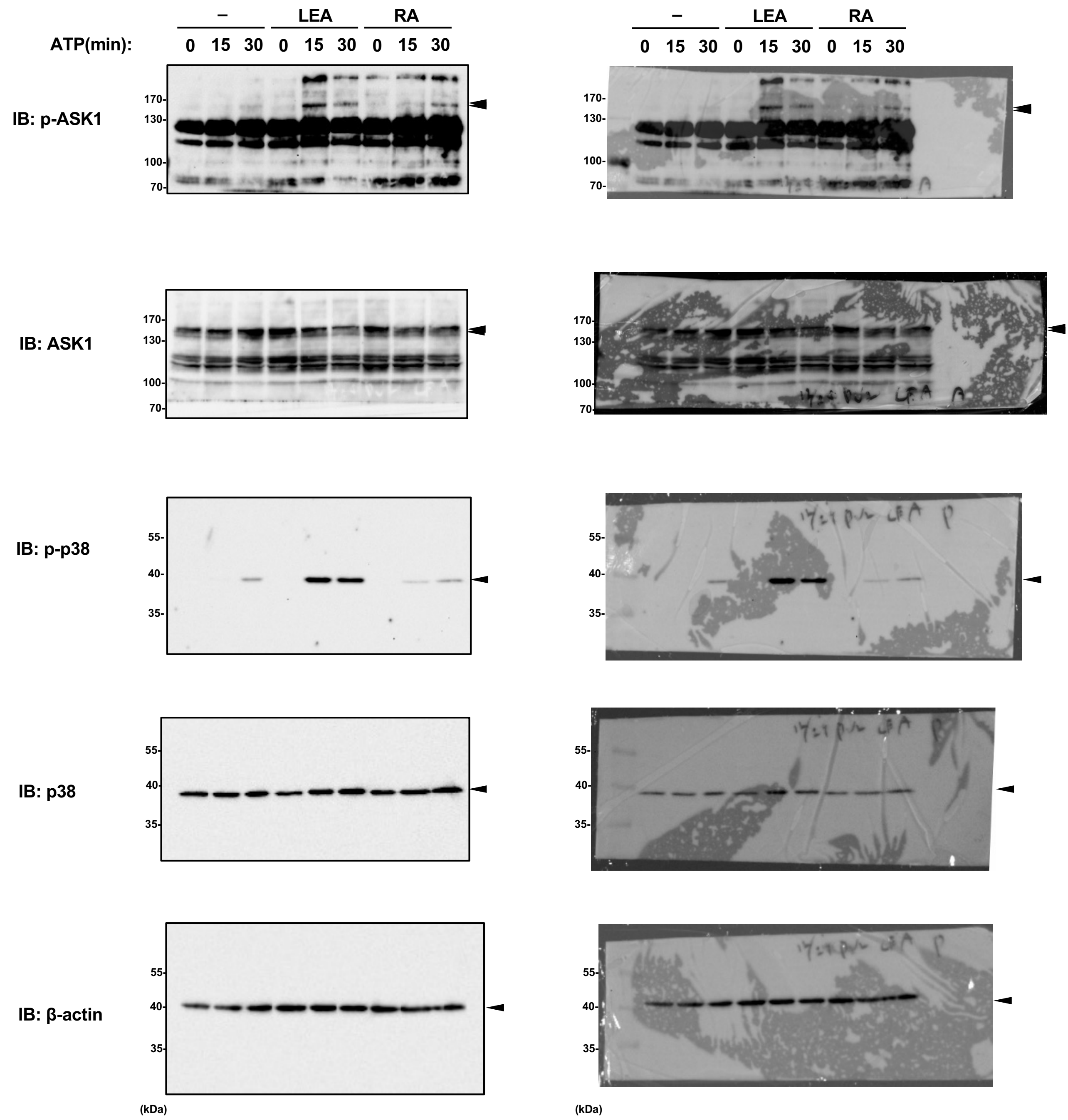

Hirata et al., Fig. S6.

e

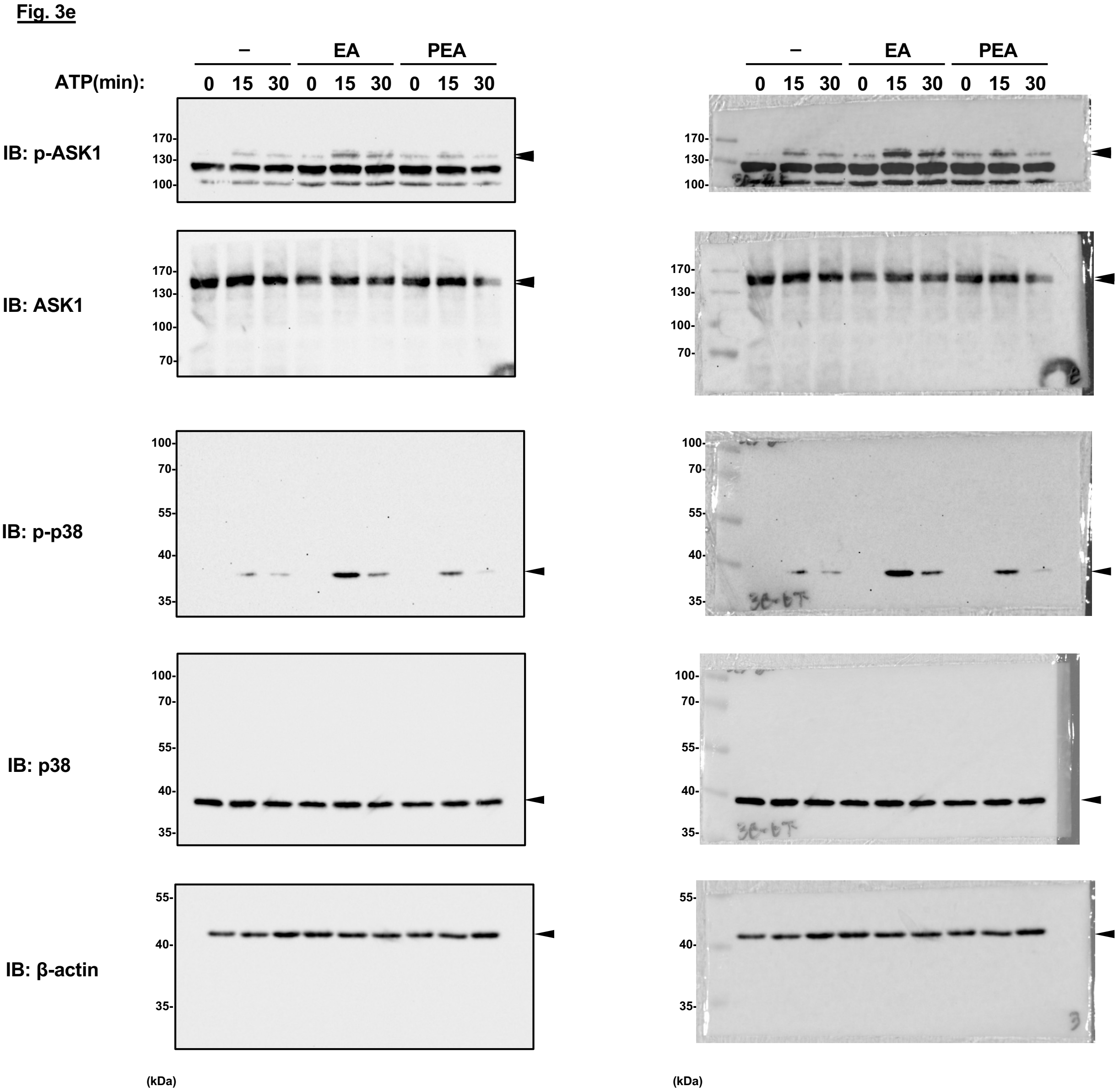

Hirata et al., Fig. S6.

f

Fig. 3f

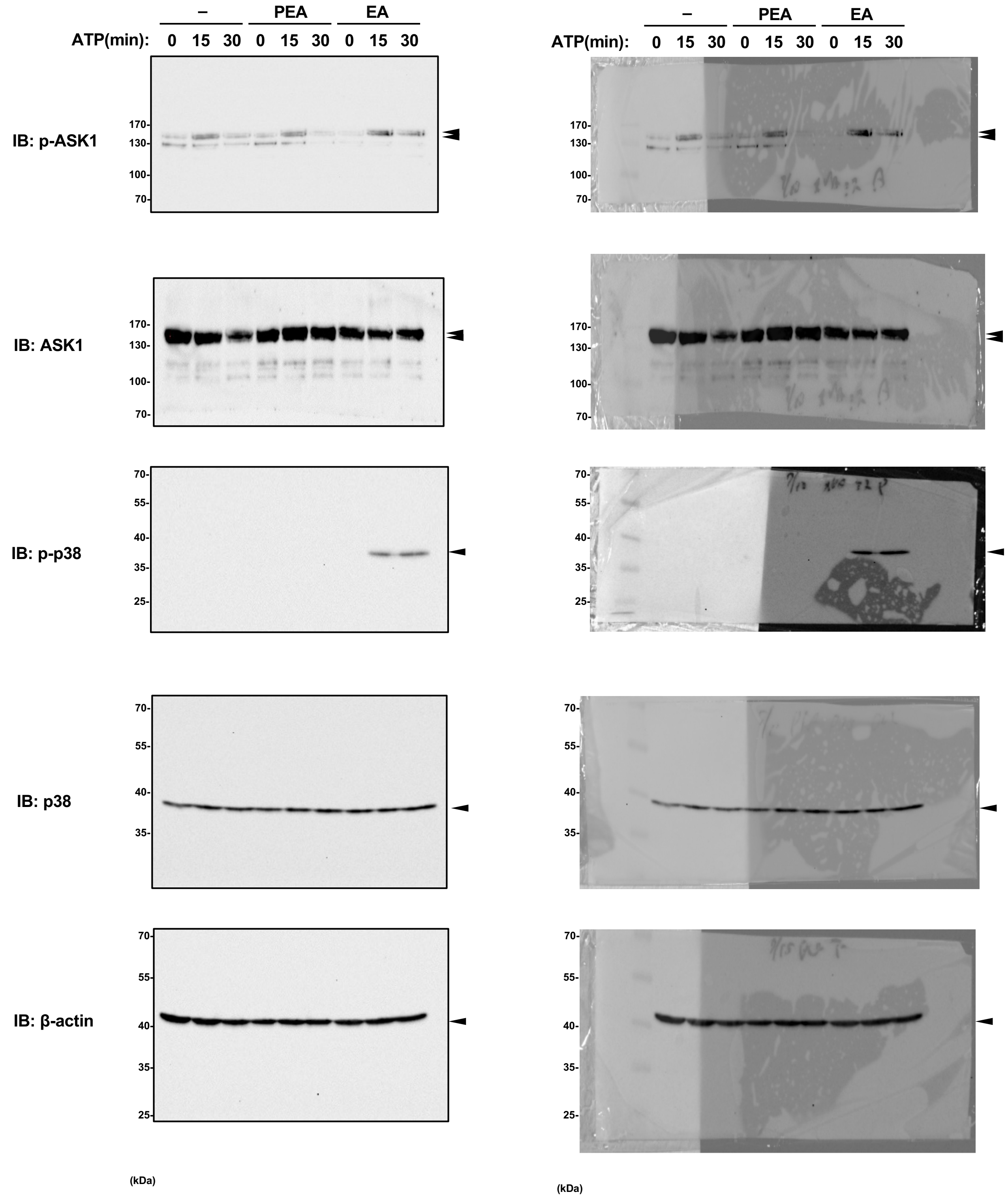

Hirata et al., Fig. S6.

g

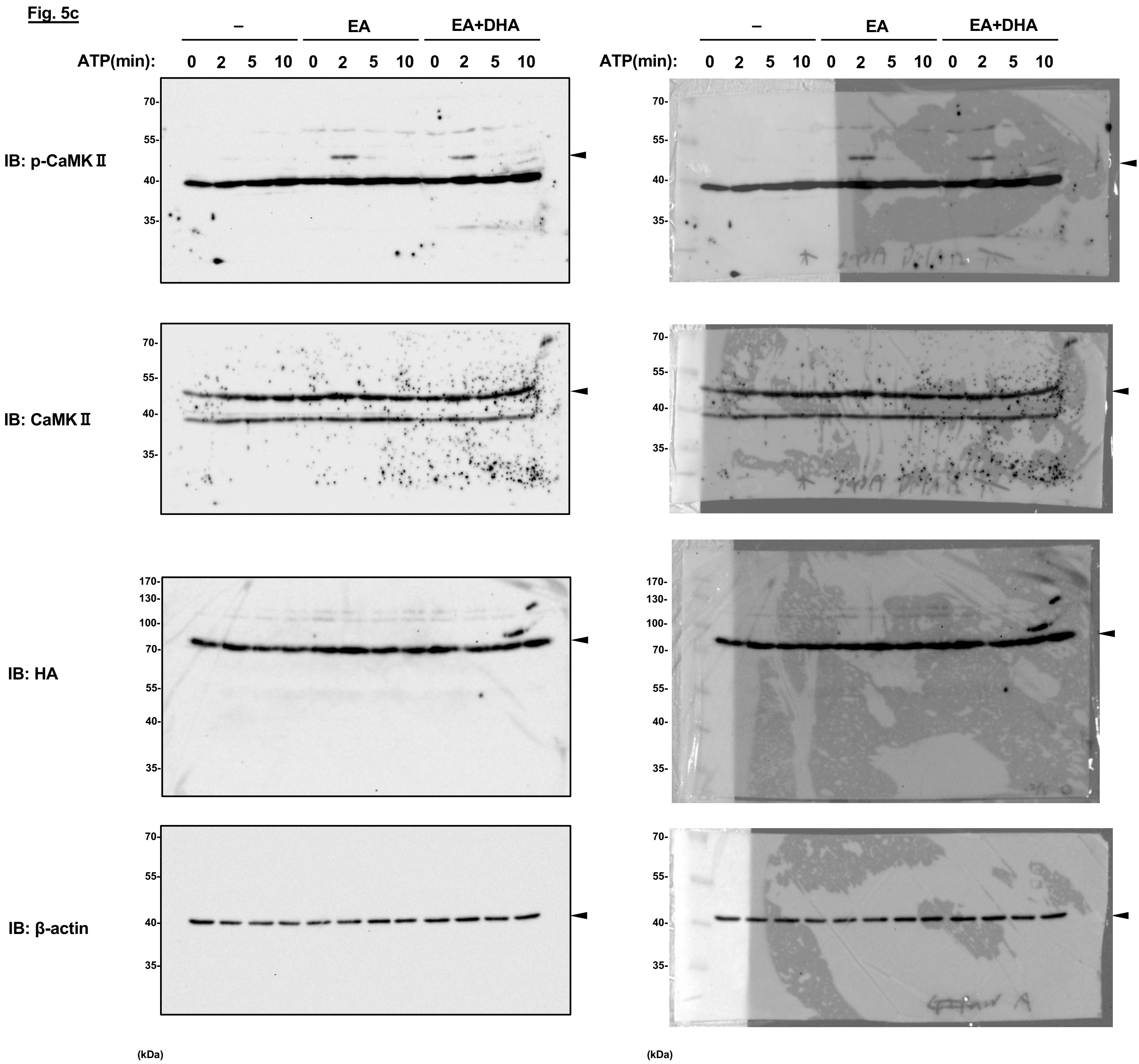

Hirata et al., Fig. S6.

h

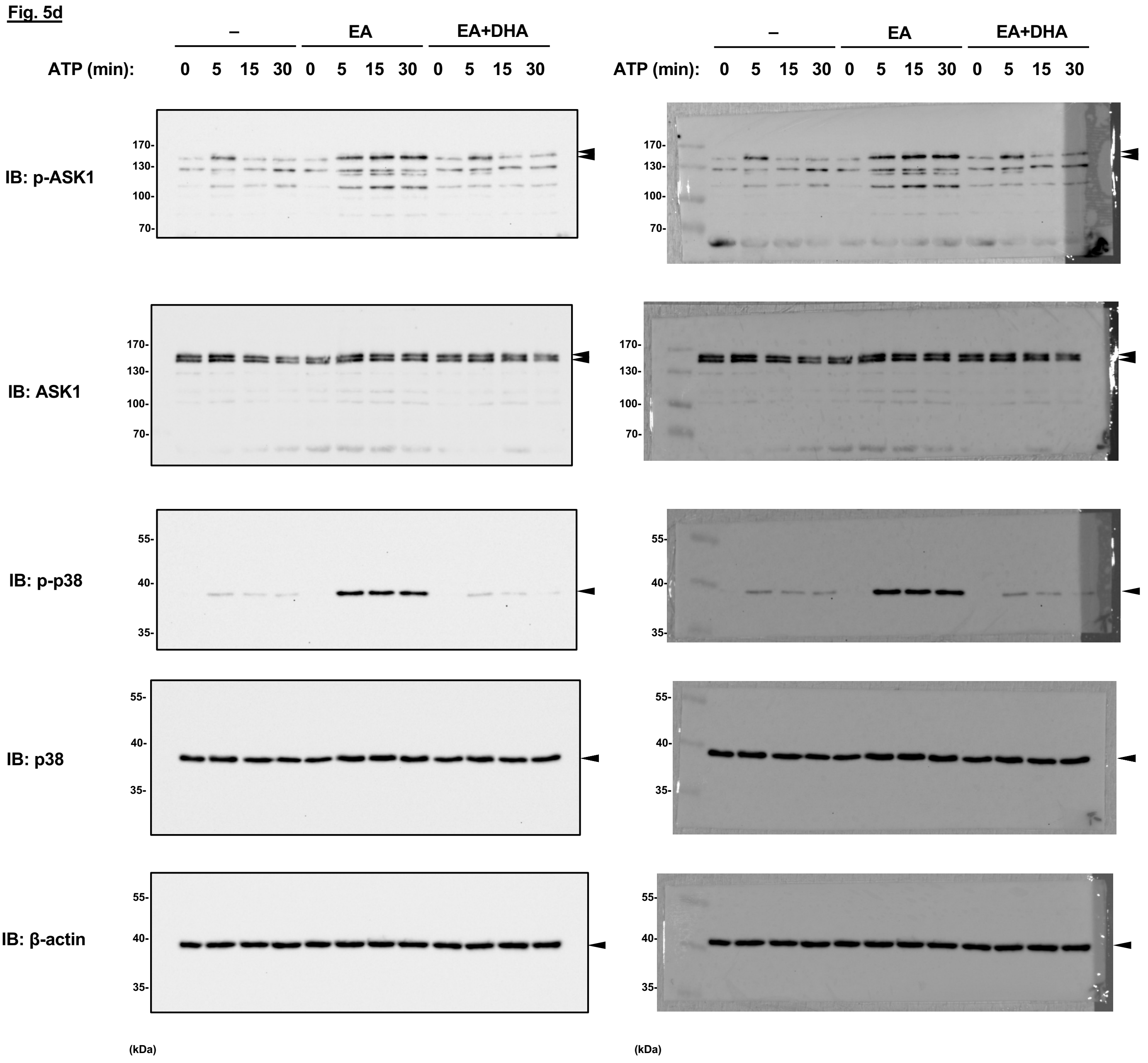
Fig. 5d
–
EA
EA+DHA
ATP (min):
0 5 15 30 0 5 15 30 0 5 15 30
IB: p-ASK1
IB: ASK1
IB: p-p38
IB: p38
IB: β-actin
170-
130-
100-
70-
55-
40-
35-
(kDa)

i

Fig. S4a

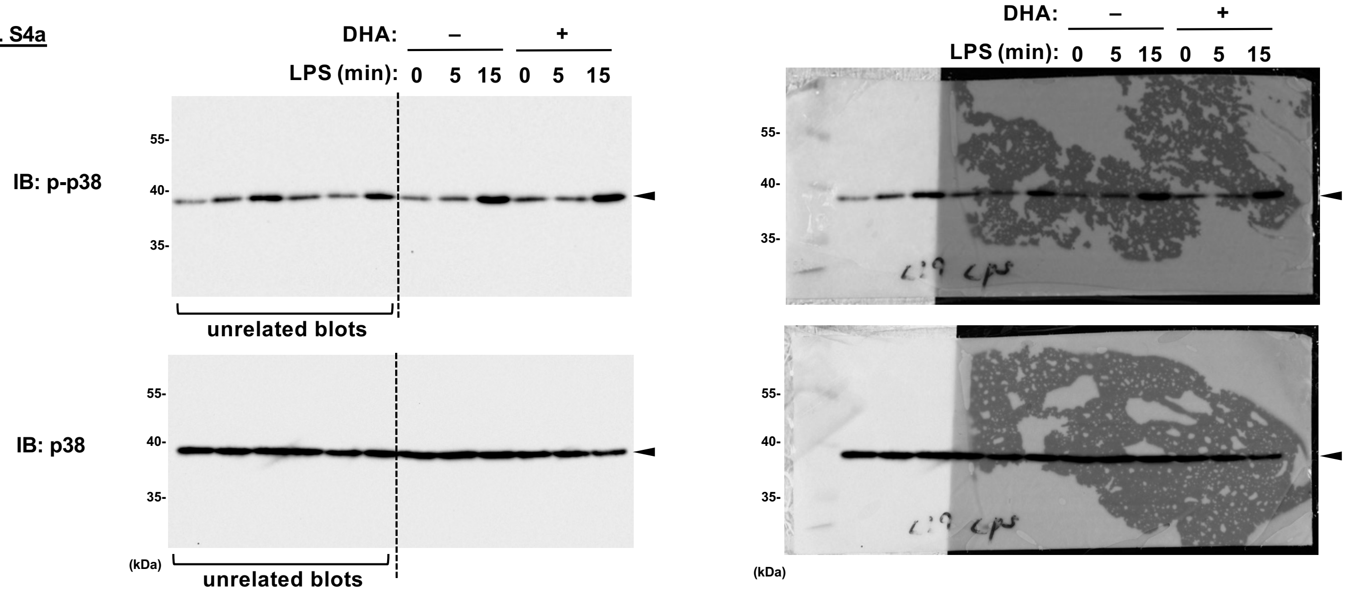

j

Fig. S4b

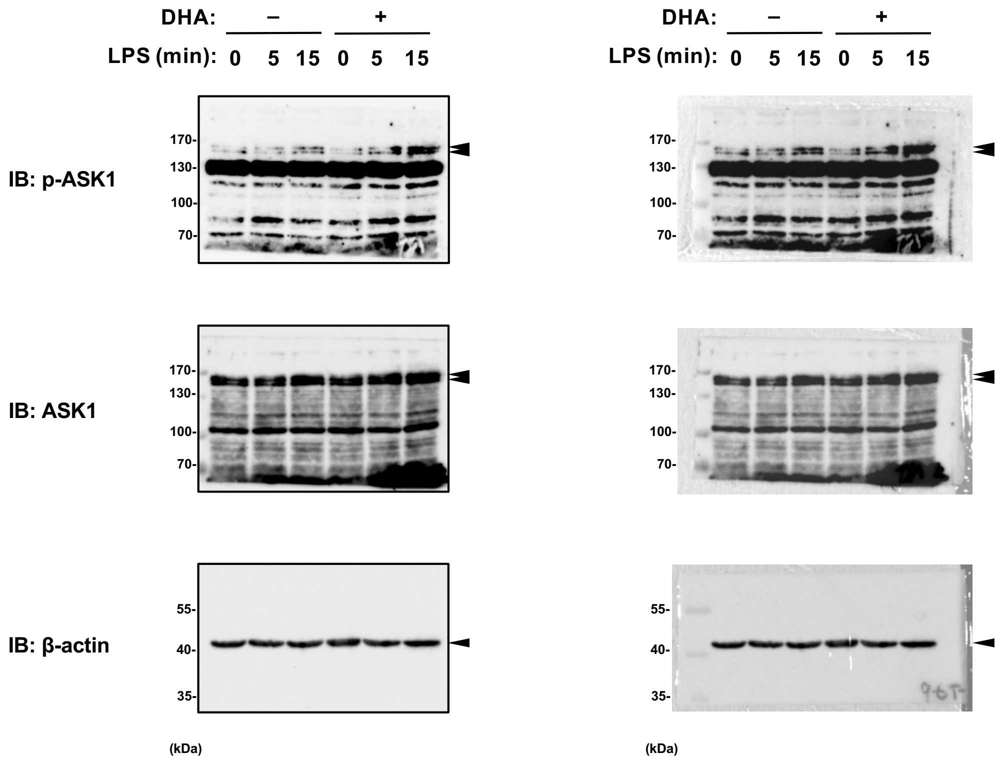

Hirata et al., Fig. S6.

k

Fig. S4c

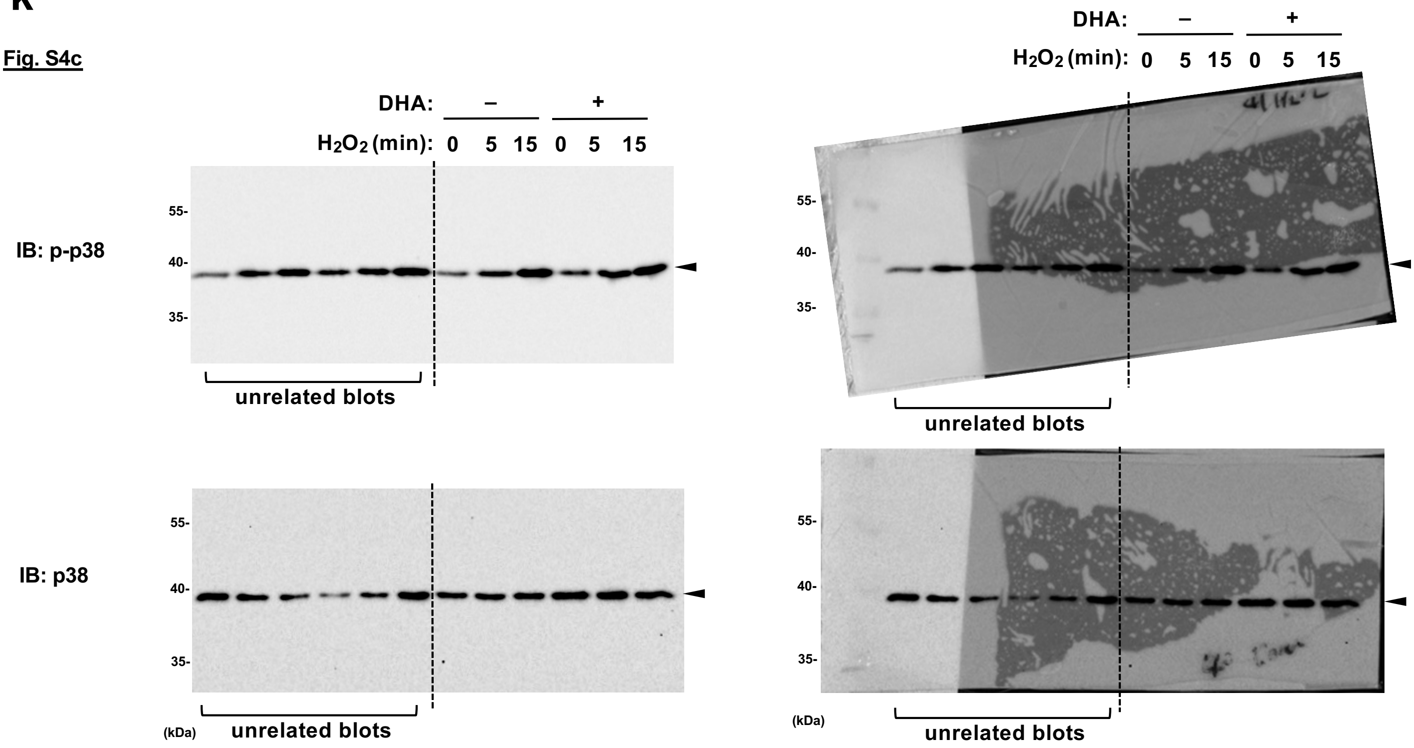

l

Fig. S4d

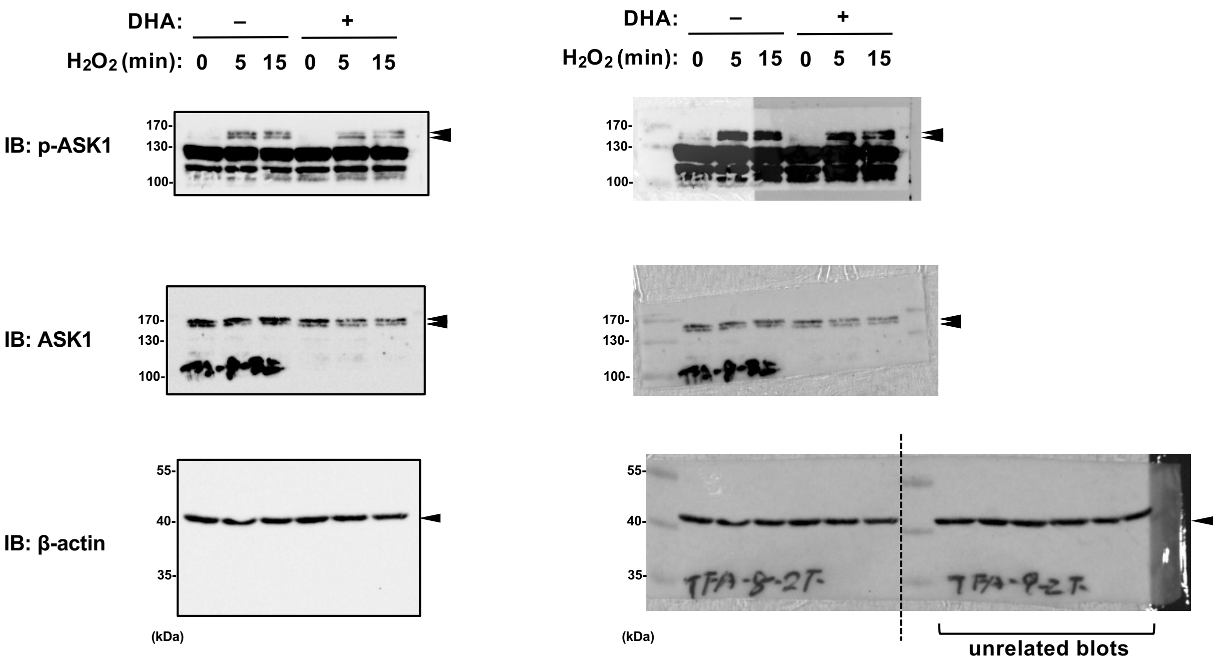

Supplementary Figure 6. Full scans of immunoblot data

(a-h) Uncropped images of Fig. 3a (a), Fig. 3b (b), Fig. 3c (c), Fig. 3d (d), Fig. 3e (e), Fig.3f (f), Fig. 5c (g), Fig. 5d (h), Fig. S4a (i), Fig. S4b (j), Fig. S4c (k) and Fig. S4d (l).

Hirata et al., Fig. S6.
